# Identification of FOXO1 as a geroprotector in human synovium through single-nucleus transcriptomic profiling

**DOI:** 10.1093/procel/pwad060

**Published:** 2023-12-13

**Authors:** Feifei Liu, Yi Lu, Xuebao Wang, Shuhui Sun, Huize Pan, Min Wang, Zehua Wang, Weiqi Zhang, Shuai Ma, Guoqiang Sun, Qun Chu, Si Wang, Jing Qu, Guang-Hui Liu

**Affiliations:** State Key Laboratory of Membrane Biology, Institute of Zoology, Chinese Academy of Sciences, Beijing 100101, China; Sports Medicine Department, Beijing Jishuitan Hospital, Capital Medical University, Beijing 100035, China; State Key Laboratory of Stem Cell and Reproductive Biology, Institute of Zoology, Chinese Academy of Sciences, Beijing 100101, China; University of Chinese Academy of Sciences, Beijing 100049, China; State Key Laboratory of Membrane Biology, Institute of Zoology, Chinese Academy of Sciences, Beijing 100101, China; Institute for Stem Cell and Regeneration, Chinese Academy of Sciences, Beijing 100101, China; Beijing Institute for Stem Cell and Regenerative Medicine, Beijing 100101, China; National Laboratory of Biomacromolecules, Institute of Biophysics, Chinese Academy of Sciences, Beijing 100101, China; State Key Laboratory of Stem Cell and Reproductive Biology, Institute of Zoology, Chinese Academy of Sciences, Beijing 100101, China; Division of Life Sciences and Medicine, School of Life Sciences, University of Science and Technology of China, Hefei 230001, China; State Key Laboratory of Stem Cell and Reproductive Biology, Institute of Zoology, Chinese Academy of Sciences, Beijing 100101, China; University of Chinese Academy of Sciences, Beijing 100049, China; CAS Key Laboratory of Genomic and Precision Medicine, Beijing Institute of Genomics, Chinese Academy of Sciences and China National Center for Bioinformation, Beijing 100101, China; State Key Laboratory of Membrane Biology, Institute of Zoology, Chinese Academy of Sciences, Beijing 100101, China; Institute for Stem Cell and Regeneration, Chinese Academy of Sciences, Beijing 100101, China; Beijing Institute for Stem Cell and Regenerative Medicine, Beijing 100101, China; State Key Laboratory of Membrane Biology, Institute of Zoology, Chinese Academy of Sciences, Beijing 100101, China; State Key Laboratory of Stem Cell and Reproductive Biology, Institute of Zoology, Chinese Academy of Sciences, Beijing 100101, China; Institute for Stem Cell and Regeneration, Chinese Academy of Sciences, Beijing 100101, China; Beijing Institute for Stem Cell and Regenerative Medicine, Beijing 100101, China; The Fifth People’s Hospital of Chongqing, Chongqing 400062, China; The Fifth People’s Hospital of Chongqing, Chongqing 400062, China; Advanced Innovation Center for Human Brain Protection and National Clinical Research Center for Geriatric Disorders, Xuanwu Hospital Capital Medical University, Beijing 100053, China; Aging Translational Medicine Center, International Center for Aging and Cancer, Beijing Municipal Geriatric Medical Research Center, Xuanwu Hospital, Capital Medical University, Beijing 100053, China; State Key Laboratory of Stem Cell and Reproductive Biology, Institute of Zoology, Chinese Academy of Sciences, Beijing 100101, China; University of Chinese Academy of Sciences, Beijing 100049, China; Institute for Stem Cell and Regeneration, Chinese Academy of Sciences, Beijing 100101, China; Beijing Institute for Stem Cell and Regenerative Medicine, Beijing 100101, China; State Key Laboratory of Membrane Biology, Institute of Zoology, Chinese Academy of Sciences, Beijing 100101, China; University of Chinese Academy of Sciences, Beijing 100049, China; Institute for Stem Cell and Regeneration, Chinese Academy of Sciences, Beijing 100101, China; Beijing Institute for Stem Cell and Regenerative Medicine, Beijing 100101, China; Advanced Innovation Center for Human Brain Protection and National Clinical Research Center for Geriatric Disorders, Xuanwu Hospital Capital Medical University, Beijing 100053, China; Aging Translational Medicine Center, International Center for Aging and Cancer, Beijing Municipal Geriatric Medical Research Center, Xuanwu Hospital, Capital Medical University, Beijing 100053, China

**Keywords:** aging, single-nucleus RNA sequencing, synovium, FOXO1, senescence

## Abstract

The synovium, a thin layer of tissue that is adjacent to the joints and secretes synovial fluid, undergoes changes in aging that contribute to intense shoulder pain and other joint diseases. However, the mechanism underlying human synovial aging remains poorly characterized. Here, we generated a comprehensive transcriptomic profile of synovial cells present in the subacromial synovium from young and aged individuals. By delineating aging-related transcriptomic changes across different cell types and their associated regulatory networks, we identified two subsets of mesenchymal stromal cells (MSCs) in human synovium, which are lining and sublining MSCs, and found that angiogenesis and fibrosis-associated genes were upregulated whereas genes associated with cell adhesion and cartilage development were downregulated in aged MSCs. Moreover, the specific cell-cell communications in aged synovium mirrors that of aging-related inflammation and tissue remodeling, including vascular hyperplasia and tissue fibrosis. In particular, we identified forkhead box O1 (FOXO1) as one of the major regulons for aging differentially expressed genes (DEGs) in synovial MSCs, and validated its downregulation in both lining and sublining MSC populations of the aged synovium. In human FOXO1-depleted MSCs derived from human embryonic stem cells, we recapitulated the senescent phenotype observed in the subacromial synovium of aged donors. These data indicate an important role of FOXO1 in the regulation of human synovial aging. Overall, our study improves our understanding of synovial aging during joint degeneration, thereby informing the development of novel intervention strategies aimed at rejuvenating the aged joint.

## Introduction

Various joints, including knee, hip, elbow, and shoulder, form connections between bones that allow for movement. Within the joint, the synovium, a connective soft-tissue membrane lining the joint capsules, not only provides structural support but also facilitates joint movements by lubricating and nourishing adjacent tissues, thereby contributing to the maintenance of joint homeostasis (Bhattaram and Chandrasekharan, 2017; Chou et al., 2020). Recently, the synovium has garnered increased attention owing to its proposed role as a nexus of pathological changes in joint diseases (Frank-Bertoncelj and Gay, 2014; Sanchez-Lopez et al., 2022). Despite its critical importance to joint health, our understanding of synovial aging-associated manifestations and the underlying mechanisms remains limited.

Histological changes observed in synovial tissue have been correlated with synovial aging. For example, chronic low grade inflammation, a characteristic feature of aged joints observed in the aged synovium (Robinson et al., 2016), may contribute to the disruption of normal tissue structure and function, leading to tissue damage and degeneration (Sanchez-Lopez et al., 2022;Geng et al., 2023). Other phenotypic traits associated with inflammation have also been reported in the aged synovium, such as the accumulation of advanced glycation end products (AGEs) and aggravated oxidative stress (Li et al., 2021). Moreover, the amount of hyaluronic acid molecules in the synovial fluid decreases during aging, compromising its ability to support cushioning and lubrication (Dahl and Husby, 1985). In addition, the synovial lymphatic system, responsible for removing catabolic factors from joints, is impaired during aging. Similarly, studies focusing on joint diseases indicate that particular molecular changes, such as elevated levels of collagenases and gelatinases, formation of immune complexes involving HLA molecules, and aberrant VEGF-C/VEGFR-3 signaling, are associated with inflammation and tissue remodeling across various disease conditions (Lin et al., 2023; Onuora, 2023). Notably, certain synoviocytes undergo a senescent state during which they release various molecules that contribute to inflammation and tissue remodeling within the synovial tissue of patients with synovitis (Bhattaram and Chandrasekharan, 2017).

As a specialized connective tissue, the synovium is composed of two layers, the lining and the sublining layers, and consists of different types of synoviocytes and immune cells that are embedded in a meshwork of extracellular matrix (ECM) (Culemann et al., 2019; Nygaard and Firestein, 2020; Orr et al., 2017; Pasquali-Ronchetti et al., 1992; Wei et al., 2020). These different cell types are likely to undergo marked changes during synovial aging, however, few studies to date focused on characterizing cell type-specific phenotypes and molecular changes associated with synovial aging.

Herein, we established the first single-nucleus transcriptomic atlas for subacromial synovial tissues from young and aged individuals. Through this dataset, we identified differentially expressed genes (DEGs) specific to each cell type, as well as gene regulatory networks associated with human synovial aging. Specifically, we found that silencing of the transcription factor FOXO1 was associated with numerous changes in different intercellular and intracellular inputs that regulate aging-related changes. Using human embryonic stem cell-derived synoviocyte-like mesenchymal stromal cells, we confirmed the role of FOXO1 in cellular senescence. Collectively, our study provides valuable insights into human synovial aging at a single-cell resolution and lays a foundation for the development of new therapeutic strategies targeting joint degeneration and related diseases.

## Results

### Age-related manifestations in human subacromial synovium

**Figure 1.**
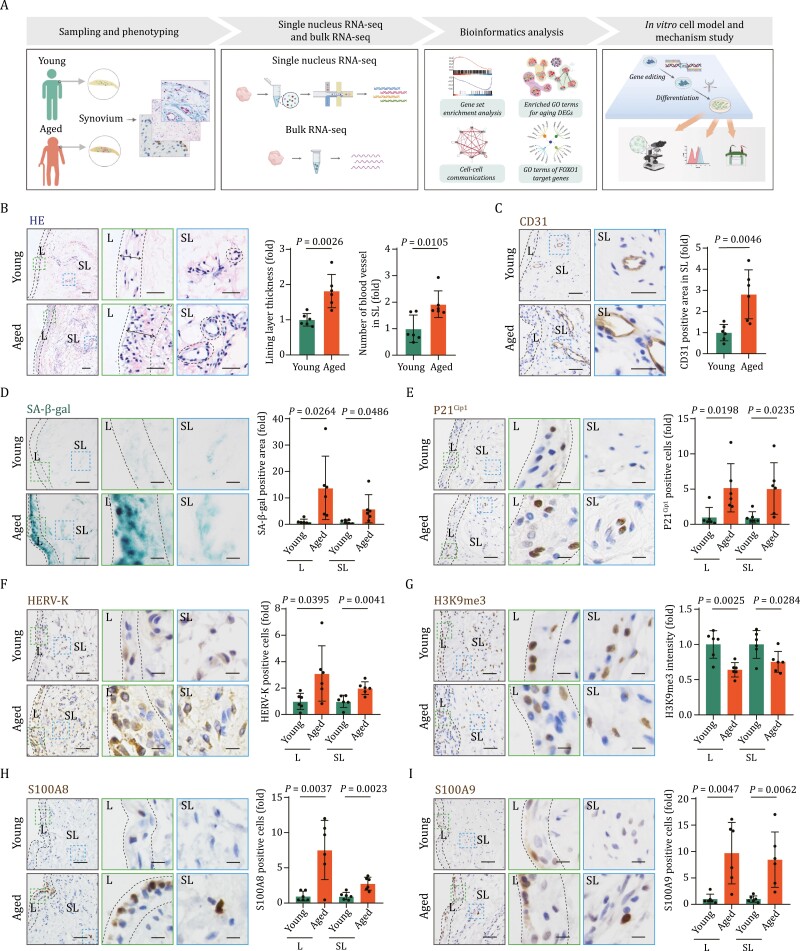
Characterizing aging-related changes in human subacromial synovium. (A) Schematic diagram of the experimental design aimed at deciphering the cellular and molecular regulatory network involved in the aging of human synovium. (B) HE staining of young and aged human synovial tissues. Left, representative images, scale bars, 50 μm and 10 μm (zoomed-in images). Right, quantitative analysis of lining layer thickness and number of blood vessel. Double-headed arrows indicate lining layer thickness. *n* = 6 individuals per group. (C) Immunohistochemistry (IHC) staining of CD31 in young and aged human synovial tissues. Left, representative images, scale bars, 50 μm and 10 μm (zoomed-in images). Right, CD31 positive area in SL region is quantified as fold changes (aged vs. young). *n* = 6 individuals per group. (D) SA-β-gal staining of young and aged human synovial tissues. Left, representative images, scale bars, 50 μm and 10 μm (zoomed-in images). Right, SA-β-gal-positive area in the indicated region (L or SL) is quantified as fold changes (aged vs. young). *n* = 6 individuals per group. (E) IHC staining of cellular senescence-associated marker P21^Cip1^ in young and aged human synovial tissues. Left, representative images, Scale bars, 50 μm and 10 μm (zoomed-in images). Right, the number of P21^Cip1^ positive cells in the indicated region (L or SL) is quantified as fold changes (aged vs. young). *n* = 6 individuals per group. (F) IHC staining of HERV-K in young and aged human synovial tissues. Left, representative images, scale bars, 50 μm and 10 μm (zoomed-in images). Right, the number of HERV-K positive cells in the indicated region (L or SL) is quantified as fold changes (aged vs. young). *n* = 6 individuals per group. (G) IHC staining for H3K9me3 in young and aged human synovial tissues. Left, representative images, scale bars, 50 μm and 10 μm (zoomed-in images). Right, the intensity of H3K9me3 in the indicated region (L or SL) is quantified as fold changes (aged vs. young). *n* = 6 individuals per group. (H) IHC staining of S100A8 in young and aged human synovial tissues. Left, representative images, scale bars, 50 μm and 10 μm (zoomed-in images). Right, the number of S100A8 positive cells in the indicated region (L or SL) is quantified as fold changes (aged vs. young). *n* = 6 individuals per group. (I) IHC staining of S100A9 in young and aged human synovial tissues. Left, representative images, scale bars, 50 μm and 10 μm (zoomed-in images). Right, the number of S100A9 positive cells in the indicated region (L or SL) is quantified as fold changes (aged vs. young). *n* = 6 individuals per group. Statistical significance was assessed using two-tailed Student’s unpaired *t* tests (B–I). Data are presented as the mean ± SEM. *P* values less than 0.05 were considered statistically significant. A green border around the zoomed-in images for the lining layer (L), and a blue border around those for the sublining layer (SL).

To investigate age-related physiological changes in subacromial synovium using histological analysis, we collected young and aged human synovial tissues from the “floor” of subacromial bursa that is in close proximity to the supraspinatus tendon (Fig. 1A; Table S1). When we made a comparison for different anatomical regions of the subacromial synovium, including the lining layer and sublining layer, we identified distinct alterations in the aged synovium.

At the structural level, blood vessel formation increased with age in the sublining layer of synovium, whereas intimal hyperplasia increased specifically in the lining layer (Fig. 1B and 1C). When we examined indicators of cellular senescence in young and aged synovium, we found that both the lining and sublining layers of aged synovium had a higher percentage of senescence-associated β galactosidase (SA-β-gal) positive areas (Fig. 1D). Similarly, cells positive for cellular senescence marker p21^Cip1^ and human endogenous retrovirus-K (HERV-K) increased in the aged synovium (Fig. 1E and 1F) (Liu et al., 2023a; Zhou et al., 2023), indicating accumulation of senescent cells in subacromial synovium during aging. The loss of heterochromatin and the reduction of heterochromatin-associated proteins HP1γ has been linked to cellular senescence and the onset of age-related diseases (Bi et al., 2020; Diao et al., 2021; Liang et al., 2021; Pal and Tyler, 2016; Tsurumi and Li, 2012). In line with these observations, we found that heterochromatin marker H3K9me3 and heterochromatin-associated protein HP1γ were downregulated in cells within lining and sublining layers of aged synovium (Figs. 1G and S1A). Senescent cell accumulation is known to be associated with pro-inflammatory processes in various organs (Zhang et al., 2021, 2023b;Liu et al., 2023a; Sun et al., 2022a), and we further investigated inflammation in the aged synovium. We observed an increased number of infiltrated immune cells in aged synovial tissues, as indicated by increased proportions of CD45-positive immune cells (Fig. S1B). Consistently, the expression of S100A8 and S100A9, which are typically expressed in activated neutrophils or macrophages in response to inflammatory stimuli (Zheng et al., 2022), was augmented in aged synovium (Fig. 1H and 1I). Collectively, our histological analysis revealed multifaceted aging-associated changes in the subacromial synovium of aged individuals.

### Transcriptomic profiling of young and aged human subacromial synovium.

**Figure 2.**
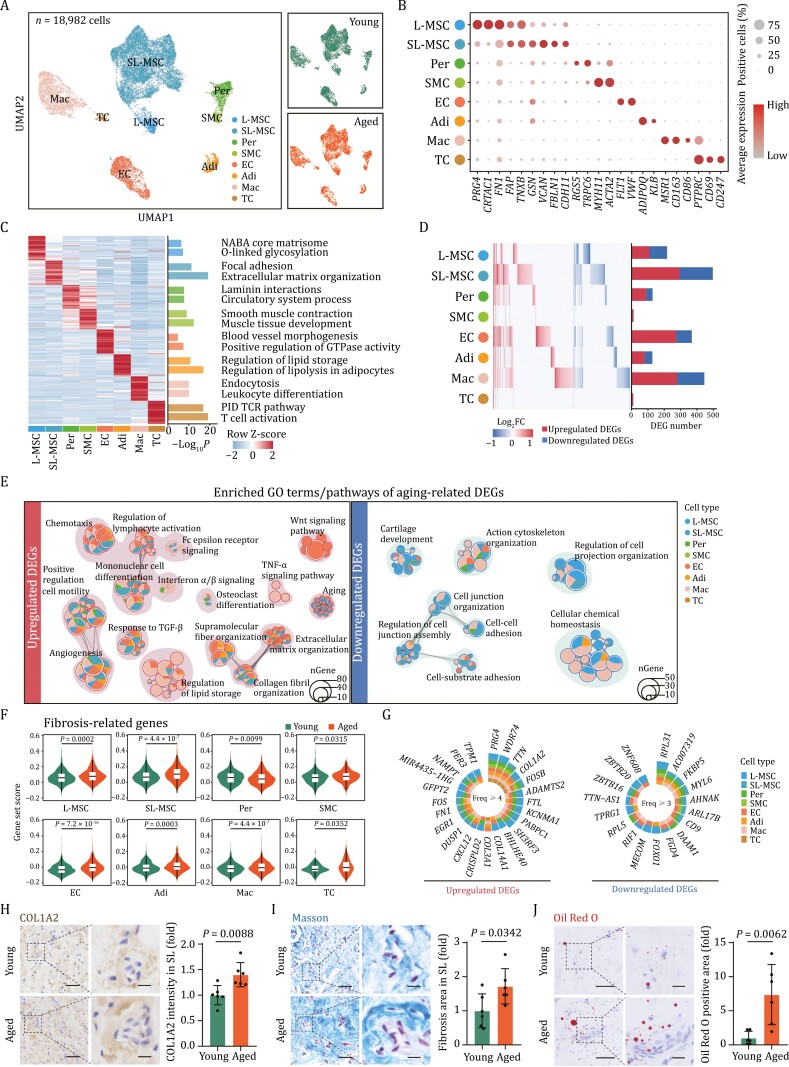
Single-nucleus transcriptomic profiling of young and aged human synovium. (A) Uniform manifold approximation and projection (UMAP) plot showing the distribution of different cell types in young and aged human synovial tissues. Distinct cell types are depicted with different colors. L-MSC, lining layer mesenchymal stromal cell; SL-MSC, sublining layer mesenchymal stromal cell; Per, pericyte; SMC, smooth muscle cell; EC, endothelial cell; Adi, adipocyte; Mac, macrophage; TC, T cell. Same UMAP visualization split by distinct age group was shown on the right. (B) Dot plot showing the expression of representative marker genes for each cell type in the synovium. (C) Heatmap showing the expression profiles of top 50 marker genes of different cell types in young and aged human synovial tissues with their enriched functional annotations on the right. (D) Heatmap and bar plot showing the Log_2_FC (left) and number (right) of aging-related DEGs. Heatmap showing the Log_2_FC of aging-related DEGs across synovial cell types. Each row represents one cell type and each column represents one aging DEGs. Bar plot showing the number of upregulated and downregulated aging DEGs across different cell types. (E) Network plots showing the enriched GO terms/pathways of upregulated DEGs (left) and downregulated DEGs (right) across all cell types during human synovial aging. The dot size indicates the number of DEGs enriched in corresponding pathways. (F) Violin plots showing the gene set score of fibrosis-related genes across different cell types of young and aged human synovial tissues. (G) Circular plots showing upregulated (left) and downregulated DEGs (right) shared by at least four or three cell types. The color key indicates different cell types. (H) IHC staining of COL1A2 in young and aged human synovial tissues. Left, representative images, scale bars, 50 μm and 10 μm (zoomed-in images). Right, the intensity of COL1A2 is quantified as fold changes (aged vs. young). *n* = 6 individuals per group. (I) Masson staining of the synovial tissues from young and aged human. Left, representative images, scale bars, 50 μm and 10 μm (zoomed-in images). Right, the intensity of collagen is quantified as fold changes (aged vs. young). *n* = 6 individuals per group. (J) Oil Red O staining of young and aged human synovial tissues. Left, representative images, scale bars, 50 μm and 12.5 μm (zoomed-in images). Right, Oil Red O-positive area is quantified as fold changes (aged vs. young). *n* = 6 individuals per group. Statistical significance was assessed using two-tailed Student’s unpaired *t* tests (H–J). Data are presented as the mean ± SEM. *P* values less than 0.05 were considered statistically significant.

Many studies have reported the presence of distinct cell populations in the synovium, including synoviocytes, vascular cells, and immune cells (Giorgino et al., 2023). To resolve the cell type-specific transcriptional changes of human synovium during aging, we conducted single-nucleus RNA sequencing (snRNA-seq) of human subacromial synovial samples from young and aged individuals (Fig. 2A; Table S1). After stringent filtration, 18,982 high-quality single-nucleus transcriptomes with a median of 2,182 genes detected to be expressed per cell remained for subsequent analysis (Figs. 2A, S2A and S2B). Using unsupervised clustering and uniform manifold approximation and projection (UMAP) analysis, we annotated and characterized eight cell types based on the expression of classic cell type-specific marker genes (Figs. 2A, 2B and S2C). All major synovial cell types were identified in each age group, including lining-mesenchymal stromal cells (*PRG4*^+^, *CRTAC1*^+^, *FN1*^+^, L-MSC, *n* = 860 cells), sublining-mesenchymal stromal cells (*GSN*^+^, *VCAN*^+^, *FBLN1*^+^, SL-MSC, *n* = 10,566 cells), pericytes (*RGS5*^+^, *TRPC6*^+^, Per, *n* = 1,191 cells), smooth muscle cells (*MYH11*^+^, *ACTA2*^+^, SMC, *n* = 351 cells), endothelial cells (*FLT1*^+^, *VWF*^+^, EC, *n* = 2,280 cells), adipocytes (*ADIPOQ*^+^, *KLB*^+^, Adi, n = 674 cells), macrophages (*MSR1*^+^, *CD163*^+^, *CD86*^+^, Mac, *n* = 2,832 cells), and T cells (*PTPRC*^+^, *CD69*^+^, *CD247*^+^, TC, *n* = 228 cells) (Figs. 2B and S2C). Functional enrichment analysis of the top 50 cell type-specific marker genes of each cluster provided insights into their distinct physiological functions, as demonstrated by the transcriptional features related to their unique physiological functions (Fig. 2C; Table S2). Specifically, L-MSC and SL-MSC represent functionally distinct mesenchymal stromal cell types. L-MSC-expressed genes related to NABA core matrisome and O-linked glycosylation, including the specific expression of PRG4, a highly-glycosylated protein that functions as a lubricant in synovial fluid. Conversely, SL-MSC-expressed genes were associated with focal adhesion and extracellular matrix organization, such as VCAN, a key component of the extracellular matrix involved in cell attachment, growth, and migration. Overall, the above analysis of cells in both young and aged synovium provided a detailed cellular landscape, highlighting the heterogeneity and functional diversity of different mesenchymal stromal cells underlying the homeostasis of synovium.

### Cell type-specific and shared transcriptional changes during synovial aging

We next calculated aging-related DEGs (averaged | log_2_FC | > 0.25 and adjusted *P*-value < 0.05) in different cell types of human subacromial synovium. Among these cell types, SL-MSC and Mac featured the largest numbers of aging-responsive DEGs (the number of upregulated DEGs was 293, 278, and the number of downregulated DEGs was 204, 167, respectively) (Fig. 2D; Table S3). Gene Ontology (GO) and function enrichment analyses of these aging DEGs revealed that genes associated with angiogenesis, response to TGF-β, lymphocyte activation, and collagen fibril organization were commonly upregulated in most of the identified cell types in aged synovium compared with the young cells (Fig. 2E). To further evaluate the fibrotic state in aged synovium, we conducted gene set score analysis for fibrosis-related gene, and observed an elevation in gene expression levels in most of the identified cell types, particularly in SL-MSC (Figs. 2F and S2D). Additionally, the expression of fibrosis markers, such as *COL1A2*, was enhanced in six out of the eight identified cell types (Fig. 2G). Immunohistochemistry staining for COL1A2 and Masson’s trichrome staining further confirmed the heightened fibrosis in both lining and sublining layers of aged synovium, with fibrotic changes being particularly prominent in the sublining layer (Fig. 2H and 2I). Specifically, genes enriched in FCERI signaling, TNF-α signaling, and regulation of lipid storage were upregulated in Mac (Fig. 2E). In line with the gene expression changes, we observed fatty infiltration as a histological change associated with synovial aging (Fig. 2J). Moreover, the expression of genes related to the Wnt signaling pathway was particularly upregulated in aged ECs, which aligns with the observed angioplasia in aged synovium (Fig. 2E). Additionally, consistent with the histological findings of accumulated senescent cells in aged synovium (Fig. 1D and 1E), gene set score analysis demonstrated a general increase in the expression of senescence-associated secretory phenotype associated gene across various cell types, especially in L-MSC and Mac (Fig. S2E and S2F). Bulk RNA sequencing of the synovial samples further confirmed activation of FCERI-mediated NF-κB activation and immune response as observed in the snRNA-seq analysis (Fig. S2G).

Conversely, downregulated genes shared across different synovial cell types were mainly involved in cell junction organization, cellular chemical homeostasis, and cartilage development (Fig. 2E). Notably, *RPL31*, a gene encoding for a ribosomal protein, was identified as a high-frequent downregulated DEG in six of the eight identified cell types (Fig. 2G). Although RPL31 was reported to be important in regulation of osteogenic differentiation (Peng et al., 2022), and was identified as one of the top dysregulated genes in the synovium of rheumatoid arthritis (RA) patients (Ramezankhani et al., 2021), the exact role of RPL31 in synovial aging or diseases remains unexplored.

**Figure 3.**
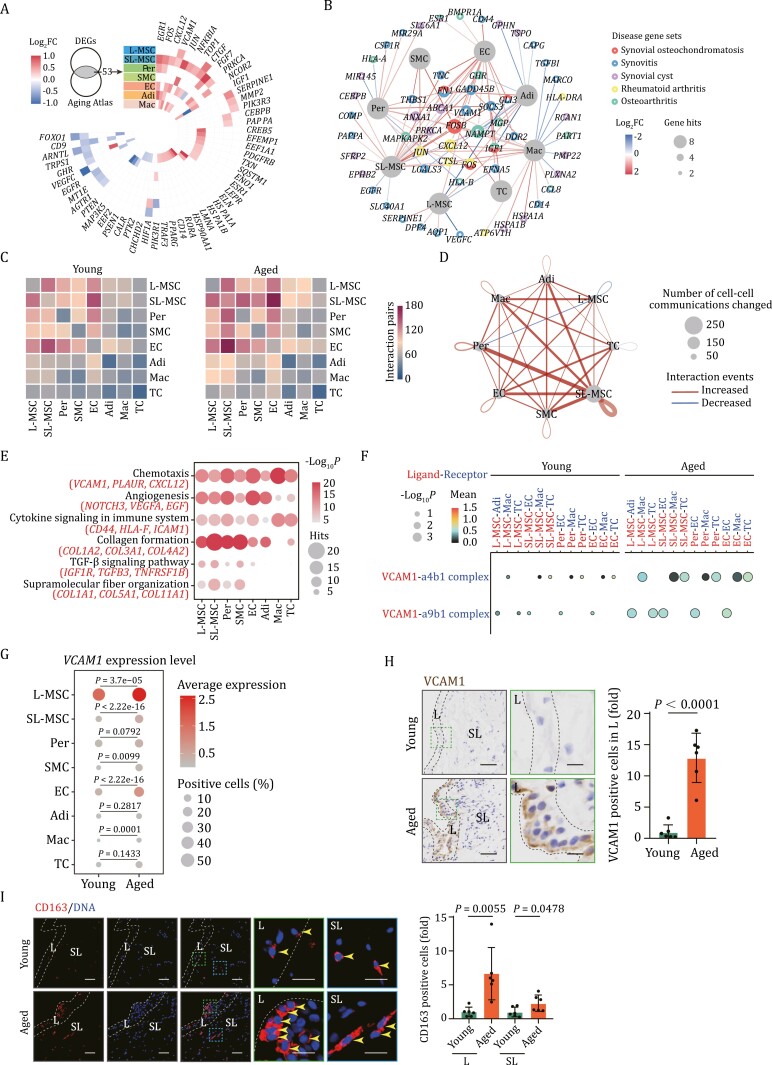
Cell-type specific transcriptional changes during human synovial aging. (A) Heatmap showing the aging DEGs annotated in aging atlas database across different cell types. (B) Network plot showing the correlation of aging-related DEGs with synovial disease gene sets. Each node represents one gene, and the node size indicates the number of cell types shared by DEGs. The color of lines indicates the Log_2_FC of DEGs across different cell types. (C) Heatmaps showing the number of interaction pairs between indicated cell types of human synovium in young and aged groups. The color key indicates the interaction counts. (D) Network plot showing the cell–cell interaction pairs changed across different cell types during synovial aging. The node size of cell types indicates the number of cell–cell interaction pairs changed. The color of lines indicates the changing directions of interaction events across different cell types. (E) Dot plot showing the representative enriched functional annotations for increased cell–cell interaction pairs during synovial aging. (F) Dot plot showing VCAM1-related cell-cell interaction pairs in indicated cell types across young and aged groups. The size of the dots represents the −log_10_*P* value and the color key indicates the mean value of expression levels. (G) Dot plot showing the expression level of VCAM1 across different cell types during synovial aging. (H) IHC staining of VCAM1 in young and aged human synovial tissues. Left, representative images, scale bars, 50 μm and 10 μm (zoomed-in images). Right, the number of VCAM1 positive cells in the L region is quantified as fold changes (aged vs. young). *n* = 6 individuals per group. (I) Immunofluorescent staining of CD163 in young and aged human synovial tissues. Left, representative images, scale bars, 50 μm and 20 μm (zoomed-in images). Right, the number of CD163-positive cells in the indicated region (L or SL) is quantified as fold changes (aged vs. young). Yellow arrows indicate CD163 positive cells. *n* = 6 individuals per group. Statistical significance was assessed using two-tailed Student’s unpaired *t* tests (H and I). Data are presented as the mean ± SEM. *P* values less than 0.05 were considered statistically significant.

By conducting joint analysis of synovial aging DEGs and genes from the Aging Atlas database (Aging Atlas, 2021), we found that the gene expression signatures of the aged synovium were associated with inflammation and osteoarthritis (OA) pathogenesis (Fig. 3A). For example, the OA risk factor *EGR1* was highly expressed in most synovial cells of aged samples, while the circadian rhythm master gene *ARNTL* was downregulated in both MSC cell types. Furthermore, *VEGFC*, a potential drug target for inflamed synovium in OA, was particularly downregulated in L-MSC (Fig. 3A) (Lin et al., 2023). By further assessing genetic risk variants associated with OA heritability, we noted that *NAMPT* was upregulated in synovial cells during aging. Similarly, *FN1*, which encodes a glycoprotein present on the cell surface or in the extracellular matrix associated with synovial osteochondromatosis and synovitis, was upregulated in different synovial cell types during aging (Fig. 3B). Taken together, these data uncover a potential molecular association between aging and joint diseases.

### Core regulons for transcriptional changes in aged human synovium

To delineate intercellular programs instructing synovial aging, we conducted cell–cell communications analysis by CellPhoneDB. This analysis enabled us to examine interactions between different cell types based on paired ligand-receptor gene expression (Fig. 3C). Overall, we found that intercellular interactions among different cell types within the synovial tissue were enhanced during aging, with SL-MSCs exhibiting the most remarkable changes (Fig. 3D). When we used GO enrichment analysis to focus on intercellular interactions that increased with age, we found that these were mainly enriched in pathways related to chemotaxis, collagen formation, and angiogenesis, while those that decreased with age were mainly enriched in pathways related to cartilage development and osteoblast differentiation (Figs. 3E and S3A–C). Notably, interactions between VCAM1 and integrins (encoded by *ITGA4* and *ITGA9*), were particularly enhanced in immune cells, such as macrophages, in aged synovium (Fig. 3F). In parallel, VCAM1, which plays an important role in leukocyte chemotaxis and adherence (Agarwal and Brenner, 2006), was expressed in L-MSCs with an overwhelming abundance and was particularly upregulated in L-MSC during aging (Fig. 3G). Transmigration of monocytes into the synovium, as the initial step for synovitis, is reported to be mainly triggered by integrin activation and chemotaxis (Lowin and Straub, 2011). Therefore, this intensified interaction between VCAM1 and integrins may contribute to the adherence and transmigration of monocytes into the lining layer of synovium, initiating an inflammatory response. Consistently, we detected an approximately 10-fold increase in VCAM1-positive cells in the lining layer of aged synovium compared to younger counterparts (Fig. 3H). Moreover, numbers of macrophages also increased with age and exhibited aggregation in close proximity to L-MSCs at the lining layer of aged synovium (Fig. 3I).

**Figure 4.**
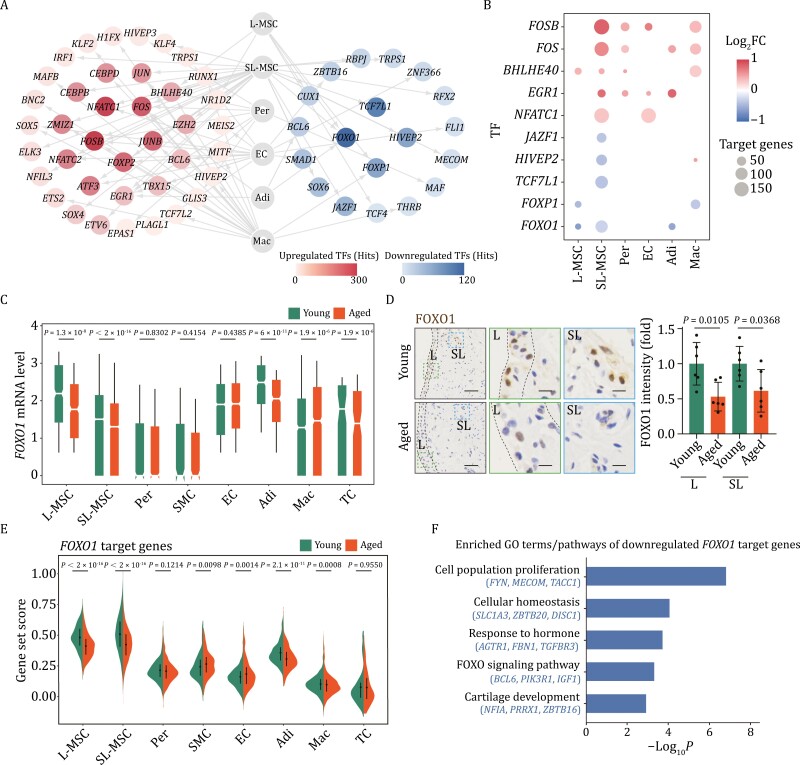
Core transcriptional regulon analysis for human synovial aging. (A) Network plot showing upregulated and downregulated core TFs across all cell types during human synovial aging. Internodes represent core TFs, and the node color of TF indicates the number of target gene hits. (B) Dot plot showing the distribution of the top core TFs across different cell types during aging. The dot size indicates the number of target gene hits. The color of the dots represents the Log_2_FC of TFs expression in the corresponding cell type. (C) Box plot showing the expression levels of *FOXO1* across different cell types during aging. (D) IHC staining of FOXO1 in young and aged human synovial tissues. Left, representative images, scale bars, 50 μm and 10 μm (zoomed-in images). Right, the intensity of FOXO1 is quantified as fold changes (aged vs. young). *n* = 6 individuals per group. (E) Violin plot showing the gene set score of FOXO1 target DEGs across each cell type of young and aged human synovial tissues. (F) Bar plot showing representative GO terms/pathways of FOXO1 target DEGs during synovial aging. Statistical significance was assessed using two-tailed Student’s unpaired *t* tests. (D) Data are presented as the mean ± SEM. *P* values less than 0.05 were considered statistically significant, a green border around the zoomed-in images for the lining layer (L), and a blue border around those for the sublining layer (SL).

Next, we applied single-cell regulatory network inference and clustering (SCENIC) analysis to identify transcription factors that potentially contribute to the transcriptional changes across different cell types in the aged subacromial synovium (Fig. 4A and 4B; Table S4). The core regulons for upregulated aging DEGs included *NFATC1* (in SL-MSC and EC), a gene known to promote osteoclast differentiation, and *SOX5* (in EC), which plays a central role in the transcription of immune response-related genes in the synovium (Fig. 4A) (Collins et al., 2023). Notably, both NFATC1 and SOX5 were reported to be upregulated in joints of patients with OA or RA (Knights et al., 2023). For the downregulated aging DEGs, we found that the top regulon was FOXO1, the expression of which was decreased in the majority of synovial cells, including L-MSC and SL-MSC (Fig. 4C). The downregulation of FOXO1 in both lining layer and sublining layer of aged synovium was validated through immunostaining (Fig. 4D). Further GO and function enrichment analysis revealed that genes associated with cartilage development and response to hormone, as FOXO1 target genes, were also downregulated during synovial aging (Fig. 4E and 4F). These data indicated that the downregulation of FOXO1 may serve as a mechanism contributing to the observed degeneration in aged synovium. Notably, FOXO1 is acknowledged for its role in repressing *VCAM1* expression (Bertin et al., 2015), and the elevated expression of VCAM1 in aged synovial MSCs may be linked to the reduced FOXO1 expression.

### Depletion of FOXO1 accelerated cellular senescence in hMSCs

Previous studies have demonstrated the downregulation of FOXO1 in aged intervertebral discs (Alvarez-Garcia et al., 2017, 2018) and OA-affected cartilage (Akasaki et al., 2014; Yue et al., 2022). However, the role of FOXO1 in synovial aging has never been studied. To explore the potential regulatory role of FOXO1 in synovial cells, we knocked out FOXO1 in human embryonic stem cells (hESCs) using transcription activator-like effector nuclease (TALEN)-mediated homologous recombination gene editing (Fig. S4A). Western blot analysis confirmed FOXO1 deficiency (Fig. S4B), while genome-wide copy number variation (CNV) analysis demonstrated that the genome was stable in *FOXO1*^–/–^ hESCs, and the karyotype was also normal (Fig. S4C and S4D). Furthermore, morphology and expression of pluripotency markers including NANOG, SOX2, and OCT4 in *FOXO1*^–/–^ hESCs were comparable to those in *FOXO1*^+/+^ hESCs (Fig. S4E). In addition, we did not detect any difference in cellular proliferation ability between *FOXO1*^+/+^ and *FOXO1*^–/–^ hESCs through Ki67 immunofluorescence staining (Fig. S4F). Altogether, these data indicated that FOXO1 is dispensable for the maintenance of pluripotency in hESCs.

**Figure 5.**
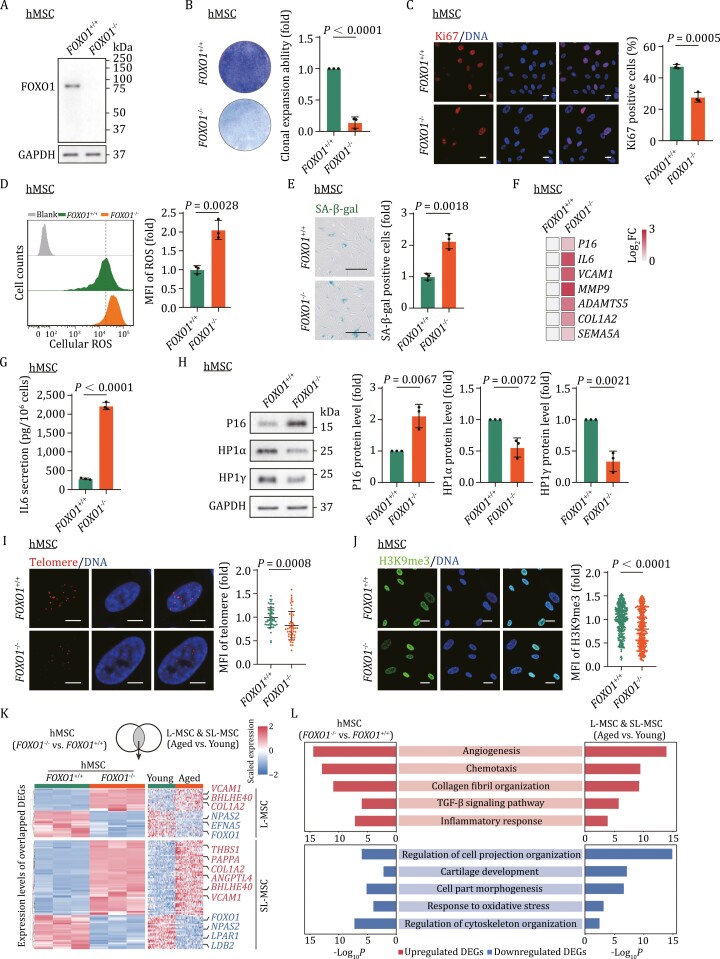
Depletion of ***FOXO1*** accelerated cellular senescence in hMSC. (A) Western blot analysis of the FOXO1 in the *FOXO1*^+/+^ and *FOXO1*^–/–^ hMSCs, GAPDH was used as the loading control. (B) Clonal expansion analysis of the *FOXO1*^+/+^ and *FOXO1*^–/–^ hMSCs. Left, representative images. Right, cell density is quantified as fold changes (*FOXO1*^–/–^ vs. *FOXO1*^+/+^), *n* = 3 independent experiments per group. (C) Immunofluorescence analysis of Ki67 in the *FOXO1*^+/+^ and *FOXO1*^–/–^ hMSCs. Left, representative images. Scale bar, 20 μm. Right, Quantification of Ki67 positive cells, *n* = 3 biological replicates per group. (D) Flow cytometric analysis of cellular ROS levels by H2DCFDA staining in the *FOXO1*^+/+^ and *FOXO1*^–/–^ hMSCs. Left, representative images. Right, the mean fluorescence intensity (MFI) of ROS is quantified as fold changes (*FOXO1*^–/–^ vs. *FOXO1*^+/+^), *n* = 3 biological replicates per group. (E) SA-β-gal staining of the *FOXO1*^+/+^ and *FOXO1*^–/–^ hMSCs. Left, representative images, Scale bar, 50 μm. Right, SA-β-gal positive cells are quantified as fold changes (*FOXO1*^–/–^ vs. *FOXO1*^+/+^), *n* = 3 biological replicates per group. (F) RT-qPCR analysis for the expression of *P16*^*INK4a*^, *IL6*, *VCAM1,* and extracellular matrix-related genes in *FOXO1*^+/+^ and *FOXO1*^–/–^ hMSCs, *n* = 3 biological replicates per group. (G) Enzyme-linked immunosorbent assay (ELISA) analysis of IL6 secretion in the culture medium of the *FOXO1*^+/+^ and *FOXO1*^–/–^ hMSCs. *n* = 3 biological replicates per group. (H) Western blot analysis of P16 ^INK4a^, HP1α, and HP1γ in the *FOXO1*^+/+^ and *FOXO1*^–/–^ hMSCs at P9. GAPDH was used as the loading control. Band intensity is quantified as fold changes (*FOXO1*^–/–^ vs. *FOXO1*^+/+^), *n* = 3 independent experiments per group. (I) DNA-FISH detection of telomere in the *FOXO1*^+/+^ and *FOXO1*^–/–^ hMSCs. Left, representative images, scale bar, 10 μm. Right, MFI of telomere per hMSC is quantified as fold changes (*FOXO1*^–/–^ vs. *FOXO1*^+/+^), *n* = 100 cells per group. (J) Immunofluorescence analysis of H3K9me3 in *FOXO1*^+/+^ and *FOXO1*^–/–^ hMSCs. Left, representative images, scale bar, 20 μm. Right, MFI of H3K9me3 per hMSC is quantified as fold changes (*FOXO1*^–/–^ vs. *FOXO1*^+/+^), *n* = 300 cells per group. (K) Heatmaps showing the expression profiles of overlapped DEGs between snRNA-seq (L-MSC and SL-MSC) and bulk RNA-seq between *FOXO1*^–/–^ and *FOXO1*^+/+^ hMSCs. (L) Bar plot showing representative pathways of upregulated and downregulated DEGs overlapped between snRNA-seq (L-MSC & SL-MSC) and bulk RNA-seq between *FOXO1*^+/+^ and *FOXO1*^–/–^ hMSCs. Statistical significance was assessed using two-tailed Student’s unpaired *t*-test. Data are presented as the mean ± SEM. *P* values less than 0.05 were considered statistically significant.

Next, we differentiated *FOXO1*^+/+^ and *FOXO1*^–/–^ hESCs into synoviocyte-like human mesenchymal stromal cells (hMSCs). These hMSCs displayed the typical markers of MSCs and exhibited the expression of a specific set of genes associated with fibroblast-like synoviocytes (Fig. S5A) (Li et al., 2019, 2020a, 2020b), thereby serving as a cellular model to investigate the functional and molecular regulations in synoviocytes. We assessed the expression of FOXO1 in *FOXO1*^+/+^ hMSCs at both early (EP) and late passages (LP). In replicative senescent hMSCs, we observed a decrease in FOXO1 expression (Fig. S5B), which aligns with our observation in L-MSCs and SL-MSCs within aged synovium (Fig. 4D). Next, we verified the ablation of FOXO1 in *FOXO1*^–/–^ hMSCs by western blot analysis (Fig. 5A). *FOXO1*^–/–^ hMSCs exhibited retarded cellular growth, compromised clonal expansion ability, decreased Ki67-positive cells, and decreased percentage of S-phase cells (Figs. 5B, 5C, S5C and S5D). In addition, the deficiency of FOXO1 markedly elevated reactive oxygen species (ROS) levels (Fig. 5D) and resulted in an increase in SA-β-gal positive cells (Fig. 5E). Next, we detected upregulated expression of a panel of SASP-related genes, such as IL6 (Fig. 5F), and accordingly, elevated IL6 secretion in *FOXO1*^–/–^ hMSCs (Fig. 5G). Concomitantly, we also observed typical indicators of cellular senescence, including increased P16^INK4a^ expression and shortened telomere in *FOXO1*^–/–^ hMSCs (Fig. 5F, 5H, and 5I) (Aging Biomarker Consortium et al., 2023; López-Otín et al., 2013). Moreover, FOXO1 deficiency led to the loss of heterochromatin marker H3K9me3 in hMSCs (Fig. 5J). Concurrently, western blot analysis revealed decreased expression of heterochromatin-associated protein HP1α and HP1γ in *FOXO1*^–/–^ hMSCs (Fig. 5H). Altogether, these findings indicate that deficiency of FOXO1 promoted accelerated senescence of hMSCs.

In concert with the aforementioned observations, our RNA-seq data demonstrate that FOXO1 deficiency induced a transcriptomic signature resembling the transcriptomic changes in human synovium during aging. There are 103 aging DEGs in L-MSC or SL-MSC that were also changed in hMSCs upon FOXO1 deficiency, including 62 upregulated genes, and 41 downregulated genes (Fig. 5K). Moreover, upregulated DEGs in FOXO1-deficient hMSC and aged synovium were enriched in angiogenesis, chemotaxis, and inflammatory response pathway, whereas downregulated genes were enriched in the regulation of cell projection organization and cartilage development (Fig. 5K and 5L). Focusing on the common DEGs shared between snRNA-seq data of L-MSC ∩ SL-MSC (aged vs. young) and bulk RNA-seq data of hMSCs (*FOXO1*^–/–^ vs. *FOXO1*^+/+^), we identified a total of 16 genes that exhibited a strong correlation with *FOXO1* expression (Fig. S5E and S5F). Within this set, further motif analysis revealed that *SDK1* gene possessed a typical FOXO1 binding site located within 500 bp upstream of its transcription start site (Fig. S5G).

**Figure 6.**
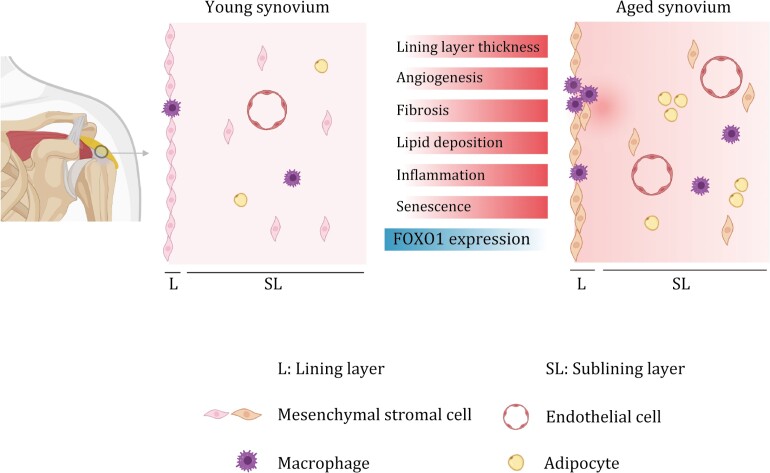
Working model. Schematic chart showing the histological changes and transcriptomic signatures of human synovial aging.

Taken together, our study demonstrates that FOXO1, the downregulation of which is predicted to be a prominent regulatory hub of aging-related transcriptomic changes in the synovium, may play an important role in synoviocytes, safeguarding cells from senescence and secretion of inflammatory cytokines and chemokine factors (Fig. 6).

## Discussion

The synovium, an essential component of joints, plays a crucial role in maintaining joint homeostasis. During aging, the synovium becomes inflamed and thicker, leading to tissue remodeling and joint conditions that ultimately result in intense shoulder pain, negatively impacting the quality of life for affected individuals (Wei et al., 2020; Aging Biomarker et al., 2023). However, our current knowledge of synovial aging and its underlying mechanisms remain limited. In this study, we conducted a single-nucleus transcriptomic analysis of subacromial synovial tissues from young and aged individuals to investigate age-related changes at the cellular and molecular levels. In line with the distinct alterations detected by histological analysis, such as angioplasia, intimal hyperplasia, cellular senescence, and immune cell infiltration in the aged synovium, our snRNA-seq data analysis demonstrated that aging in synovium leads to changes in cell expression programs, with SL-MSC and macrophages showing the largest number of DEGs. Functional enrichment analysis of these DEGs revealed upregulation of genes linked to angiogenesis, fibrosis, and inflammation across most cell types of the aged synovium. Furthermore, we identified FOXO1 as a core regulon of synovial aging and validated in human embryonic stem cell-derived synoviocyte-like mesenchymal stromal cells that FOXO1 plays a role in cellular senescence. Collectively, our study provides comprehensive insights into cellular and molecular changes associated with synovial aging, helping to pave the way for the development of innovative therapeutic strategies for joint degeneration and related diseases.

To date, cell atlas research on the synovium has predominantly focused on pathologic joints, such as OA and RA, leaving a comprehensive molecular investigation into synovial aging lacking. Here, we demonstrated that disease-associated histological phenotypes commonly observed in both OA and RA, such as cell hyperplasia, tissue fibrosis, stromal vascularization, and macrophage infiltration (Bartok and Firestein, 2010; McInnes and Schett, 2017; Smolen et al., 2016), are also evident in aged synovium. Consistently, in distinct immune and stromal cell populations within aged synovium, we observed upregulation of chemokines (*CXCL12* and *CCL8*), and inflammatory cytokines (*NFKBIA* and *VCAM1*). In parallel, macrophage subsets showed an abundance of the inflammation-triggering alarmins S100A8/9 in RA synovium (Alivernini et al., 2020). S100A8/9 is known to act as chemoattractants for neutrophils and to stimulate monocytes and fibroblasts to produce inflammatory factors (Zhang et al., 2023a), such as TNF and IL-6, thereby serving as positive feedback factors for synovial inflammation (Migita et al., 2017; Wang et al., 2018). Notably, our snRNA-seq data revealed that both synovial mesenchymal stromal cells and macrophages are the cell types exhibiting the most prominent transcriptional responses to aging, and based on the histological analysis, they also closely interact with each other in the synovial lining layer. Since monocyte adherence and transmigration into the synovium are commonly recognized as initial steps in the development of synovitis, this may further contribute to the elevated inflammation and increased thickness of lining layer. However, whether the underlying mechanism is related to FOXO1 awaits further investigations. Our study underscores the importance of these cell populations and their interplay as a potential connection between synovial aging and its associated diseases.

Furthermore, an important finding arising from this research is that FOXO1 acts as an important regulator for the aging of synovial cells. As an evolutionarily conserved family of transcription factors, FOXO proteins play crucial roles in developmental processes, aging, and longevity (Kahn, 2015). The disruption of FOXO1 expression or its activity is known to contribute to numerous age-related diseases, such as OA (Sun et al., 2022b; Wang et al., 2020), osteoporosis (Rached et al., 2010) and muscle atrophy (Oyabu et al., 2022). Mechanistically, FOXO1 participates in a diverse array of biological processes associated with aging, including DNA repair, anti-oxidative defense, cell cycle regulation, metabolic balance, and maintenance of proteostasis (Cai et al., 2022; Kousteni, 2011;Huang et al., 2022). Our data illustrates a discernible downregulation of FOXO1 in aged synovium, particularly in synovial MSCs. This observation is consistent with the reduced protein level of FOXO1 in senescent hMSCs derived from hESCs. It is noteworthy that FOXO1 expression can be downregulated by the inflammatory milieu (Ludikhuize et al., 2007), therefore, the observed downregulation of FOXO1 in aged synovial tissues is likely attributable to the inflammatory environment. On the cellular level, our investigations demonstrated that silencing FOXO1 in hMSCs led to a range of aging-related phenotypes, including cellular senescence, increased expression of SASP factors, and heightened oxidative stress. Interestingly, we identified *SDK1* as a potential target gene of FOXO1, and its expression was found to be downregulated in FOXO1 deficient hMSCs, as well as in aged L-MSC and SL-MSC. Notably, previous research has reported reduced *SDK1* expression in the synovium of patients with systemic lupus erythematosus (Hubbard et al., 2020), suggesting its potential involvement in synovial disorder. Further studies are warranted to elucidate its functional role in cellular senescence and synovial aging.

In summary, our work complements existing transcriptomic landscapes and also expands our knowledge about the specific changes that occur during human synovial aging. The distinct biology of the different synovial cell populations we describe here has important implications for therapies targeting joint diseases and synovial aging. Additionally, we identify FOXO1 as a novel gatekeeper of human synovial cell senescence. In all, these findings provide a foundation for further research on synovial aging and related joint diseases.

## Materials and methods

### Patients and specimens

Human synovial samples were collected from young (36.6 ± 0.9 years old) and aged (71.8 ± 1.2 years old) patients with a partial tear in their rotator cuff. All of them were treated with arthroscopic surgery for their shoulder pathology, whereas patients with previous surgery were excluded from the study. The sample collection in this study was approved by the Ethics Committee of Beijing Jishuitan Hospital (Approval No: Beijing Jishuitan Hospital 201611-03).

### Cell culture

*FOXO1* ^+/+^ hESCs (H9 hESCs, WiCell Research) and *FOXO1*^–/–^ hESCs were seeded on mitomycin C (MMC) inactivated mouse embryonic fibroblast (MEF) in hESCs medium, in which including Dulbecco’s modified Eagle medium/Nutrient Ham’s F12 (DMEM/F12, Thermo Fisher Scientific) containing 20% Knockout Serum Replacement (Thermo Fisher Scientific), 5 μmol/L β-mercaptoethanol (Thermo Fisher Scientific), 2 mmol/L GlutaMAX (Thermo Fisher Scientific), 10 ng/mL bFGF (Joint Protein Central), 1% penicillin/streptomycin (Thermo Fisher Scientific), 0.1 mmol/L nonessential amino acids (NEAA, Thermo Fisher Scientific) at 37°C with 5% CO_2_ for further *in vitro* experiment. hMSCs derived from hESCs were cultured in minimum essential medium α (MEMα, Thermo Fisher Scientific) supplemented with 10% fetal bovine serum (Gibco, Cat# 10091-148, Lot# 22500491P), 1 ng/mL bFGF, 0.1 mmol/L NEAA and 1% penicillin/streptomycin.

### Generation of *FOXO1*^−/−^ hESCs

For the generation of *FOXO1* knockout (*FOXO1*^−/−^) hESCs, we performed two rounds of TALEN-mediated gene editing. In brief, *FOXO1*-TALEN_Left and *FOXO1*-TALEN_Right TALENs were purchased from Addgene (Plasmid #36748 and Plasmid #36749), were used to target the human *FOXO1* gene. Donor plasmids were created by combining 1.2–1.3 kb of *FOXO1* homology arms with drug-resistance cassettes (neo or puro). In the initial round of gene knockout, H9 ESCs were dissociated using TrypLE (Invitrogen) and filtered through a 40-μm cell strainer to eliminate cell clumps. Then the cells were resuspended in 1 mL MEF-conditioned medium supplemented with 10 μmol/L ROCK inhibitor. Plasmids, including 10 μg *FOXO1*-TALEN_Left, 10 μg *FOXO1*-TALEN_Right, and 30 μg neomycin-resistant donor vector were mixed with the cell suspension and prepared for electroporation. Following electroporation, cells were plated onto 100 mm dishes coated with MMC-inactivated DR4 MEF cells. G418 (50 μg/mL) was added to the medium after electroporation 2 days later. After 14 days of selection, G418-resistant clones were manually picked into 96-well plates and expanded for genotyping. Heterozygous knockout clones were identified and subjected to a subsequent round of gene knockout. In the second round of gene knockout, the same procedures were repeated using a puromycin-resistant donor vector and puromycin selection (1 μg/mL), instead of the neomycin-resistant donor vector and G418 selection.

### Generation and characterization of hMSCs

The hMSCs were generated from hESCs. Briefly, embryoid bodies derived from hESCs were seeded on 6-well plates coated with Matrigel (BD Biosciences, 354230), the cells were cultured for about 14 days in hMSCs differentiation medium, which was performed as previously reported (Liang et al., 2021;Chu et al., 2022). The differentiation medium was changed every other day until the fibroblast-like cells appeared and reached confluent. Subsequently, the cells were then transferred into hMSCs culture medium for a continuous culture. Finally, cell sorting was conducted utilizing the fluorescence activating cell sorter (FACS) system (BD FACS Influx). CD73, CD90, and CD105 triple-positive cells were collected and further characterized by surface antigen markers, including positive marker CD44, and negative markers CD34 and CD45. The following antibodies were used for FACS: anti-CD73-PE (BD Biosciences, 550257), anti-CD90-FITC (BD Biosciences, 555595), and anti-CD105-APC (BD Biosciences, 17-1057-42), anti-CD44-FITC (BD Biosciences, 550989), anti-CD34-FITC (BD Biosciences, 555821), and anti-CD45-FITC (BD Biosciences, 555482).

### CNV identification

CNV analysis was performed as previously reported (Diao et al., 2021). Briefly, genomic DNA of *FOXO1*^+/+^ and *FOXO1*^−/−^ hESCs was extracted using a DNeasy Blood & Tissue Kit (Qiagen). Then genomic DNA was subjected to ultrasonication by Covaris. DNA Library Prep Reagent Set for Illumina (NEB) was used to construct sequencing libraries. The genome was next divided into continuous 500 kb windows with read Counter, and the absolute number of reads detected in each window was calculated. The corrections of GC content and mappability were evaluated with HMMcopy.

### Clonal expansion assay

Briefly, 3,000 cells were cultured in 0.1% gelatin-coated 12-well plate, and cultured for 13 days until the cells were almost confluent. The cells were then fixed with 4% paraformaldehyde (PFA) for 20 min, cells were washed with 1× PBS for three times at room temperature (RT) and then stained with 0.2% crystal violet (Biohao, C0520) for 1 h at RT and washed with water for at least three times. The solution of 0.2% crystal violet was filtered through 0.45 μm filters before use. The relative cell density was quantified using ImageJ software.

### SA-β-gal staining

SA-β-gal staining of synovial tissue and hMSCs was performed as previously reported (Liu et al., 2023b). Briefly, 10 μm sections of human synovial tissue or hMSCs that grow in the 6-well plate were directly washed twice with 1× PBS, and fixed with 0.2% glutaraldehyde and 2% formaldehyde at RT for less than 5 min. Then, the samples were loaded with 1 mg/mL X-gal staining buffer at 37°C for 12 h. The slides of synovial tissue were then mounted with 70% glycerol. Images were taken with Olympus VS200 system.

### Masson-trichrome staining

The paraffin-embedded synovial sections were deparaffinized with xylene, followed by rehydration through a graded ethanol series and rinsing with running tap water. Masson-trichrome staining was detected based on the manufacturers’ instruction (Solarbio, G1340). Images were collected by Olympus VS200 system.

### Hematoxylin and eosin (HE) staining

Human synovial sections for histology were fixed in PFA, then subjected to dehydration, and subsequently embedded in paraffin. The paraffin-embedded synovial sections were deparaffinized with xylene and rehydrated with a graded ethanol series and water. The sections were loaded in hematoxylin for 5 min and rinsed in tap water for 5 min. Then the slides were counterstained with eosin, dehydrated with 50% alcohol, 95% alcohol, and 100% alcohol for 1 min, respectively, and cleaned with xylene. The dyed images were captured by the Olympus microscope.

### Immunohistochemistry

Paraffin sections were deparaffinized and rehydrated, and antigen was retrieved by microwave heat treatment in citrate buffer (pH 6.0) for 25 min and then penetrated with 0.3% Triton X-100 in 1× PBS for 1 h, and afterward placed in 5% blocking buffer (normal donkey serum, 017-000-021; Jackson) for 1 h at RT. Primary antibodies were incubated in a humidified chamber overnight at 4°C. Then slides were incubated with 3% H_2_O_2_ for 15 min for the inactivation of endogenous peroxidase before incubation with a secondary antibody, followed by colorimetric detection using DAB kit (ZSGB-BIO) and then counterstaining with hematoxylin for 5 min. Finally, the sections were dehydrated and mounted in neutral resinous mounting medium. Images were captured by using an Olympus VS200 system. The following antibodies were used for Immunohistochemistry: anti-CD31 (R&D Systems, AF3628), anti-P21^Cip1^ (Cell Signaling Technology, 2947), anti-ERVK7 (United States Biological, 302427), anti-H3K9me3 (Abcam, ab8898), anti-S100A8 (Abcam, ab180735), anti-S100A9 (Abcam, ab92507), anti-FOXO1 (Cell Signaling Technology, 2880), and anti-VCAM1 (Abcam, ab134047).

### Oil Red O staining

Oil Red O staining was performed according to Yang et al. and the manufacturer’s instructions (Yang et al., 2023). The working solution of Oil Red O (Sigma–Aldrich, #O1391) was prepared by diluting it from the stock solution, mixing it with water in a 3:2 ratio, and then filtering it through 0.45 μm filters to eliminate any impurities. The OCT-embedded synovial sections were loaded with Oil Red O working solution for 15 min at RT. Slides were washed three times in water without shaking, sections were then counterstained with hematoxylin for 5 min. Images were obtained using Olympus VS200 system, and area of lipid droplets was quantified using the ImageJ software.

### Immunofluorescence

The pretreatment of samples for immunofluorescence was the same as for immunohistochemistry, antigen was retrieved by microwave heat treatment in citrate buffer (pH 6.0) for 25 min. and then penetrated with 0.3% Triton X-100 in PBS for 1 h and further placed in 5% blocking buffer (normal donkey serum, 017-000-021; Jackson) for 1 h at RT. Sections were then incubated with the appropriate primary antibodies in 5% donkey serum overnight at 4°C. After additional several washes in PBS, slides were incubated with secondary antibodies in the dark for 1 h at RT, and the nuclei were labeled with Hoechst 33342 (Thermo Fisher Scientific, H3570). Finally, Images were captured using ZEISS confocal laser scanning microscope LSM900 system. The following antibodies were used for immunofluorescence: anti-CD163 (Abcam, ab182422), anti-CD45 (Abcam, ab10558), anti-Ki67 (ZSGB-Bio, ZM-0166), anti-H3K9me3 (Abcam, ab8898). anti-OCT3/4 (Santa Cruz, sc-5279), anti-SOX2 (Santa Cruz, sc-17320), and anti-NANOG (Abcam, ab21624).

### DNA fluorescence *in situ* hybridization (DNA-FISH)

Telomere DNA-FISH were carried out in accordance with established procedures in previous publication (Kishi et al., 2019). Briefly, cells were fixed with 4% (*v*/*v*) paraformaldehyde in PBS for 10 min at RT, permeabilized with 0.5% (*v*/*v*) Triton X-100 in PBS for 10 min. Cells were then rinsed in 1× PBS, incubated in 0.2 mol/L HCl for 5 min, and washed in 2× SSCT (2× SSC with 0.1% (*v*/*v*) Tween-20), followed by an incubation in prehybridization buffer (2× SSCT, 50% formamide) at 60°C for 2 h. The cells were then transferred to an ISH solution containing 2× SSCT, 10% (*w*/*v*) dextran sulfate, 50% (*v*/*v*) formamide, 0.4 mg/mL RNase A (TIANGEN, RT405-12), and 100 nmol/L telomere probe. After denaturation at 85°C for 5 min, cells were incubated overnight at 37°C in a hybridization oven. After ISH, cells were washed in 2× SSCT at 60°C three times for 10 min and in 2× SSCT at RT twice for 2 min. Cells were transferred to a fluor hybridization solution (1× PBS, 1 μmol/L fluor probe) and incubated at 37°C for 1 h, then washed in 1× PBS at RT twice for 5 min. Finally, the cells were counterstained with Hoechst 33342 (Thermo Fisher Scientific, H3570) for 5 min. Images were captured using a ZEISS LSM900 confocal microscope and were then analyzed with ImageJ software. The probes used in this study are listed in Table S5.

### Enzyme-linked immunosorbent assay (ELISA)

The assessment of IL6 levels in the culture medium of *FOXO1*^+/+^ and *FOXO1*^−/−^ hMSCs was performed with an ELISA Kit (Biolegend, 430504) following the manufacturer’s instructions. The optical density was measured at 450 nm using Synergy H1 (BioTek). The results were normalized based on cell numbers for each well.

### Cell cycle analysis

*FOXO1* ^+/+^ and *FOXO1*^−/−^ hMSCs were collected and fixed in 75% ethyl alcohol overnight at−20°C. Cells were then washed with 1× PBS and centrifuged at 1000 ×*g* for 5 min, and stained in buffer containing 0.2 mg/mL RNase A, 0.02 mg/mL propidium iodide (PI), and 0.1% Triton X-100 at 37°C for 30 min. Next, samples were directly analyzed with a LSRFortessa cell analyser (BD) and data were analyzed using the ModFit software.

### Measurement of ROS

The ROS levels was assessed using 5-(and-6)-chloromethyl-2ʹ,7ʹ-dichlorodihydrofluorescein diacetate, acetyl ester (CM-H_2_DCFDA) staining (Invitrogen, #C6827). Cells were incubated with 2.5 μmol/L CM-H2DCFDA for 20 min at 37°C with protection from light, and then analyzed by BD LSRFortessa flow cytometry.

### Western blot

*FOXO1* ^+/+^ and *FOXO1*^–/–^ hMSCs cultured in 6-well plates were lysed on ice with 120 μL of radioimmunoprecipitation assay (RIPA) buffer (Invent, IN-WB001) supplemented with protease inhibitor cocktail (EDTA-free) (Roche, 4693159001) and PhosSTOP phosphatase inhibitor (Roche, 4906837001) for 30 min, and then centrifuged at 13,500 ×*g* for 15 min at 4°C to extract the supernatant. Then, the protein concentration was measured using BCA protein quantification kit (Dingguo Changsheng Biotech, BCA02). Each sample was then subjected to SDS-PAGE separation and electrotransferred to PVDF membrane (Millipore, ISEQ00010). Subsequently, the membranes were blocked with 5% nonfat milk in PBST for 1 h at RT and then incubated with appropriate primary antibodies at 4°C overnight. After several washes, membranes were incubated with the secondary antibodies conjugated with horseradish peroxidase (HRP) at RT for 1 h. Imaging was captured with ChemiDoc XRS + system (Bio-Rad) and the data of protein band intensity were analyzed with ImageJ. The following primary antibodies were used for western blotting: anti-FOXO1 (Cell Signaling Technology, 2880), anti-P16^INK4a^ (BD Bioscience, 550834), anti-GAPDH (Santa Cruz, sc-365062), anti-HP1α (Cell Signaling Technology, 2616), and anti-HP1γ (Cell Signaling Technology, 2619).

### Reverse transcription-quantitative PCR (RT-qPCR) assay

RT-qPCR was performed on a QuantStudio™ 5 Real-Time PCR System (Applied Biosystems) using THUNDERBIRD SYBR qPCR Mix (TOYOBO). Total RNA was extracted using TRIzol, and 2 μg RNA was used for cDNA synthesis with the GoScript Reverse Transcription System (Promega) following the manufacturer’s instruction. qPCR was performed using the CFX384 Real-Time System (Bio-Rad Laboratories, Inc.). The relative expression of each gene was normalized to the GAPDH transcript. The primer sequences are listed in Table S5.

### Nuclei isolation and snRNA-seq on the 10× genomics platform

Briefly, the frozen synovial tissues were ground into powder quickly in the mortar with liquid nitrogen, and then 1.5 mL lysis buffer was added. Cell lysis buffer consisted of nuclease-free water with 0.1% Triton X-100, 5 mmol/L MgCl_2_, 25 mmol/L KCl, 10 mmol/L Tris buffer, 250 mmol/L sucrose, 0.4 U/μL RNasin Plus RNase inhibitor (Promega, N2615), 1 μmol/L dithiothreitol, 0.2 U/μL SUPERaseIN RNase inhibitor (Invitrogen, AM2694), and 1× protease inhibitor. The samples were filtered through a 40 μm cell strainer (BD Falcon), centrifuged at 500 ×*g* for 8 min at 4°C, and then samples were resuspended in 1× PBS supplemented with 0.1% BSA, 0.2 U/μL SUPERaseIN RNase inhibitor and 0.4 U/μL RNasin Plus RNase inhibitor. The nuclei were stained with Hoechst 33342 and PI, and the double-positive nuclei were sorted by FACS (BD Influx). The pooled nuclei from the same gender synovial tissues (*n* = 8) were subjected to single-nucleus capture using the 10× genomics single-Cell 3ʹ system. At least 9,000 nuclei per sample were captured following the standard 10× capture and library preparation protocol (10× genomics) and then sequenced in a NovaSeq 6000 sequencing system (Illumina, 20012866).

### Quality control and pre-processing of snRNA-seq data

The FASTQ files were mapped to the human reference genome (hg19) and generated the count matrix by Cell Ranger pipeline. Then the contamination of background mRNA in each sample was removed by the Cellbender following the recommended parameters (version 0.2.0) (Fleming et al., 2023). The filtered expression matrix was further analyzed with Seurat (V4.1.1) (Hao et al., 2021) package in R. To ensure the reliability of our data, nuclei with mitochondrial genes ratio greater than 2.5% or genes below 200 were excluded. DoubletFinder (version 2.0.3) (McGinnis et al., 2019) was used to remove doublets of the whole dataset. Finally, 18,982 high-quality nuclei with median of 2,182 gene expressions detected per cell were retained for downstream analysis.

### Integration, clustering, and identification of cell types

Seurat (version 4.1.1) was used to perform the integration and linear dimensional reduction. First, each sample dataset was normalized by the “SCTransform” function of Seurat. To identify the integration anchors, we applied the “PrepSCTIntegration” and “FindIntegrationAnchors” functions. Using these anchors, the dataset of all samples was integrated by the “IntegrateData” function. After integration, we calculated principal component analysis (PCA) dimensions with the “RunPCA” function and determined the significant PCs using the “ElbowPlot” and “DimHeatmap” functions. The top 17 PCs were applied for Uniform Manifold Approximation and Projection (UMAP) for dimension reduction to cluster cells with the “FindNeighbors” and “FindClusters” functions. Clusters were identified as distinct cell types by the expression of classical cell-type marker genes.

### Differential expression analysis

Calculation of aging-related DEGs between the aged and young samples in human synovium across different cell types were analyzed by the “FindMarkers” function of Seurat with Wilcoxon Rank Sum test. The cutoff for the aging-related DEGs was | Log_2_FC | > 0.25 and adjusted *P*-value < 0.05. The aging-related DEGs are shown in Table S3.

### Gene Ontology (GO) analysis

Metascape (version 3.5) (Zhou et al., 2019) was used to enrich GO terms/pathways of related genes. The networkD3 R package and Cytoscape (version 3.7.2) (Shannon et al., 2003) were used to visualize the results. The source of indicated gene sets is shown in Table S6.

### Transcription factor (TF) regulatory network analysis

The transcriptional core regulatory factors were predicted based on the snRNA-seq data. Transcription factor-binding motifs were identified with the GENIE3 R packages (version 1.6.0) and the RcisTarget database (version 1.6.0) of the SCENIC (version 1.1.2.2) workflow using default options (Aibar et al., 2017). Using the hg19 RcisTarget database with motifs that have genome-wide rankings, enriched transcription factor-binding motifs and candidate target genes (regulons) were identified and predicted by RcisTarget. Based on the DEGs across different cell types, GENIE3 (version 1.6.0) (Huynh-Thu et al., 2010) created gene regulatory networks. The transcriptional regulatory network was displayed by Cytoscape (version 3.7.2).

### Cell–cell communication analysis

Using CellphoneDB software (version 2.0) (Efremova et al., 2020), we performed cell–cell communication analysis. Only ligands and receptors that were expressed in more than 10% of cells in a given cell type were further evaluated. By comparing the average expression of each ligand-receptor pair among different cell types, only those with *P*-value < 0.01 were used for further prediction of cell–cell communication in various groups.

### Bulk RNA-seq analysis

The RNA-seq raw data was trimmed by Trim Galore. After quality control and adaptor trimming, the clean sequencing reads were mapped to the human reference genome (hg19) with HISAT2 software (version 2.1.0) (Kim et al., 2015). The mapped data was counted using HTSeq (version 0.13.5) (Anders et al., 2015). DEGs were identified using DESeq2 (version 1.2.4) (Love et al., 2014) with a cutoff of Benjamini–Hochberg adjusted *P*-value < 0.05 and |Log_2_FC| > 0.5. ClusterProfiler (version 4.6.0) (Wu et al., 2021) was used to perform Gene Set Enrichment Analysis. The DEGs are shown in Table S3.

### Statistical analysis

Statistical analysis was performed using GraphPad Prism 8.0.2 (GraphPad Prism Software, CA, USA). All data represent mean ± SEM. Comparisons between the two treatments were conducted using the two-tailed Student’s *t* test. For analysis of differences between the groups, one-way ANOVAs with Tukey’s or Dunnett’s multiple comparison tests were performed. *P* values less than 0.05 were considered statistically significant.

## Supplementary Material

pwad060_suppl_Supplementary_Tables_S1

pwad060_suppl_Supplementary_Figures_S1-S5

pwad060_suppl_Supplementary_Tables_S2

pwad060_suppl_Supplementary_Tables_S3

pwad060_suppl_Supplementary_Tables_S4

pwad060_suppl_Supplementary_Tables_S5

pwad060_suppl_Supplementary_Tables_S6

## Data Availability

All data associated with this study are present in the paper or the supplementary materials. The raw sequence data reported in this paper have been deposited in the Genome Sequence Archive in National Genomics Data Center, China National Center for Bioinformation, with accession number HRA005143.

## Abbreviations

Adi: adipocytes
CM-H_2_DCFDA: 5-(and-6)-chloromethyl-2ʹ,7ʹ-dichlorodihydrofluorescein diacetate, acetyl ester
CNV: copy number variation
DEGs: differentially expressed genes
DNA-FISH: , DNA fluorescence *in situ* hybridization
EC: endothelial cells
ECM: extracellular matrix
ELISA: enzyme-linked immunosorbent assay
EP: early passage
FOXO1: forkhead box O1
GO: gene ontology
GSEA: gene set enrichment analysis
HE: hematoxylin and eosin
HERV-K: human endogenous retrovirus-K
hESC: human embryonic stem cell
hMSC: human mesenchymal stromal cell
L-MSC: lining-mesenchymal stromal cells
LP: late passage
Mac: macrophages
MEF: mouse embryonic fibroblast
MFI: mean fluorescence intensity
MMC: , mitomycin C
OA: osteoarthritis
Per: pericyte
PFA: paraformaldehyde
RA: rheumatoid arthritis
RT: room temperature
RT-qPCR: reverse transcription-quantitative PCR
SA-β-Gal: senescence-associated β galactosidase;
SCENIC: single-cell regulatory network inference and clustering
SL-MSC: sublining-mesenchymal stromal cells
SMC: smooth muscle cells
snRNA-seq: single-nucleus RNA sequencing
TALEN: transcription activator-like effector nuclease;
TC: T cells
TF: transcription factor
UMAP: uniform manifold approximation and projection
