# Supplementary material for: Identification of FOXO1 as a geroprotector in human synovium through single-nucleus transcriptomic profiling
Source: Protein Cell. 2023 Dec 13;15(6):441–59. doi: 10.1093/procel/pwad060 (PMC11131031; doi:10.1093/procel/pwad060)
Supplement: pwad060_suppl_Supplementary_Figures_S1-S5 [file pwad060_suppl_supplementary_figures_s1-s5.pdf]

Supplemental materials

Figure S1. Aging-related histological changes in human subacromial synovium

(A) IHC staining of HP1γ in young and aged human synovial tissues. Left, representative images. Scale bars, 50 μm and 10 μm (zoomed-in images). Right, the intensity of HP1γ in the indicated region (L or SL) is quantified as fold changes (Aged vs. Young). $n = 6$ individuals per group.

(B) Immunofluorescence staining of CD45 in young and aged human synovial tissues. Left, representative images. Scale bars, 50 μm and 20 μm (zoomed-in images). Yellow arrows indicate CD45 positive cells of zoom-in images. Right, the number of CD45 positive cells per field of vision in SL region is quantified as fold changes (Aged vs. Young). $n = 6$ individuals per group.

Statistical significance was assessed using two-tailed Student's unpaired t test. Data are presented as the mean ± SEM. P values less than 0.05 were considered statistically significant.

Figure S2. Aging-related transcriptional changes in human synovium

(A) Bar plots showing the number of cells (left) and the percentage of reads mapped to genome (right) in each group.

(B) Box plots showing the number of genes detected (left) and the number of unique molecular identifiers (right) in each cell across different groups.

(C) UMAP plot showing the average expression of classical marker genes across different cell types in the human synovium. Color key indicates the expression level of marker genes in each cell.

(D) Ridge map showing the global distribution density of gene set score of fibrosis-related genes of young and aged human synovial tissues. The corresponding dashed line represents the median position of each group.

(E) Ridge map showing the global distribution density of gene set score of SASP of young and aged human synovial tissues.

(F) Violin plot showing the gene set score of SASP across different cell types of young and aged human synovial tissues.

(G) GSEA enrichment curves show the change of the aging-related pathway by bulk RNA-seq.

Figure S3. Changes in cell-cell interaction pairs during human synovial aging

(A) Bubble plot showing the representative enriched functional annotations for decreased cell-cell interaction pairs during synovial aging.

(B) Dot plot showing the high-frequency (frequency ≥ 5) aged-specific cell-cell interaction pairs in indicated cell types across young and aged groups. The size of the dots represents the $-\text{Log}_{10}P$ value and the color key indicates the mean value of expression levels.

(C) Dot plot showing the high-frequency (frequency ≥ 4) young-specific cell-cell interaction pairs in indicated cell types across young and aged groups. The size of the dots represents the $-\text{Log}_{10}P$ value and the color key indicates the mean value of expression levels.

Figure S4. Generation and characterization of *FOXO1*$^{-/-}$ hESCs

(A) Schematic diagram of *FOXO1* gene editing strategy using TALEN mediated homologous recombination gene editing in hESCs.

(B) Western blot analysis of the FOXO1 in *FOXO1*$^{+/+}$ and *FOXO1*$^{-/-}$ hESCs. GAPDH was used as the loading control.

(C) Copy number variation analysis of the *FOXO1*$^{+/+}$ and *FOXO1*$^{-/-}$ hESCs by whole genome sequencing.

(D) G-banded karyotyping analysis of the *FOXO1*$^{-/-}$ hESCs.

(E) Immunofluorescence images of pluripotency markers NANOG, SOX2 and OCT4 and phase-contrast images for the *FOXO1*$^{+/+}$ and *FOXO1*$^{-/-}$ hESCs. Scale bar, 25 μm

(F) Immunofluorescence analysis of Ki67 in *FOXO1*$^{+/+}$ and *FOXO1*$^{-/-}$ hESCs. Scale bar, 20 μm. The statistical analysis of Ki67 positive cells is shown on the right. n = 3 biological replicates per group.

Statistical significance was assessed using two-tailed Student's unpaired t test (F). Data are presented as the mean ± SEM.

Figure S5. Depletion of *FOXO1* accelerated hMSC senescence

(A) Western blot analysis of FOXO1 in hMSCs at EP (P3) and LP (P11), GAPDH was used as a loading control.

(B) FACS analysis of hMSC specific markers (CD44, CD73, CD90, and CD105) and hMSC irrelevant markers (CD34 and CD45) in the *FOXO1*$^{+/+}$ and *FOXO1*$^{-/-}$ hMSCs.

(C) Growth curve showing cumulative population doubling of *FOXO1*$^{+/+}$ and *FOXO1*$^{-/-}$ hMSCs. n = 3 biological replicates per group.

(D) Cell cycle analysis of *FOXO1*$^{+/+}$ and *FOXO1*$^{-/-}$ hMSCs at P9. n = 3 biological replicates per group.

(E) Plot showing the overlapped DEGs between sn-RNA seq data (L-MSC ∩ SL-MSC, aged vs. young) and bulk RNA-seq data (*FOXO1*$^{-/-}$ vs. *FOXO1*$^{+/+}$ hMSCs). The color of the gene represents the change in expression level, with red indicating upregulation and blue indicating downregulation.

(F) Scatter plots showing the expression levels of FOXO1 correlated genes and *FOXO1* based on the snRNA-seq data. In both young and aged groups, every 20 nuclei of L-MSC and SL-MSC were aggregated into a single point. The gene expression levels for each point were calculated as the average expression of the nuclei within that specific point.

(G) Table showing the NES values and motifDb data of *SDK1*, which is an overlapping gene between the FOXO1 correlated genes and the FOXO1 target genes predicted by SCENIC in L-MSC and SL-MSC. MotifDb data indicated the predicted binding sites within 500 bp upstream of the transcription start site (TSS) for the target gene.

Statistical significance was assessed using one-way ANOVAs with Dunnett's multiple comparison tests (C) or two-tailed Student's unpaired t test (D). Data are presented as the mean ± SEM. ns, not significant, $^{**}P < 0.01$, $^{***}P < 0.001$.

Supplementary Table Legends

Table S1. Sample information used in this study.

Table S2. Marker genes of different cell types identified in snRNA-seq of human synovium.

Table S3. Aging-related differentially expressed genes of bulk RNA-seq and snRNA-seq datasets.

Table S4. Core regulatory transcription factors of differentially expressed genes in human synovium during aging.

Table S5. List of primers and probes used in this study.

Table S6. Source of indicated gene sets.

Figure. S1

A

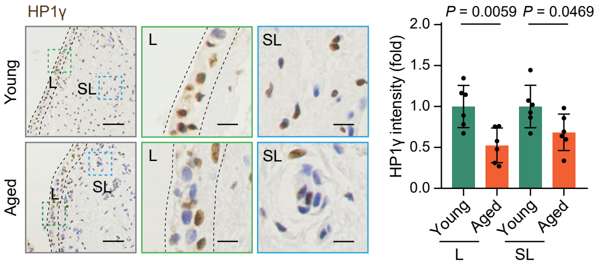
HP1γ
Young
Aged
L
SL
P = 0.0059
P = 0.0469
HP1γ intensity (fold)
2.0
1.5
1.0
0.5
0.0
Young
Aged
Young
Aged
L
SL

B

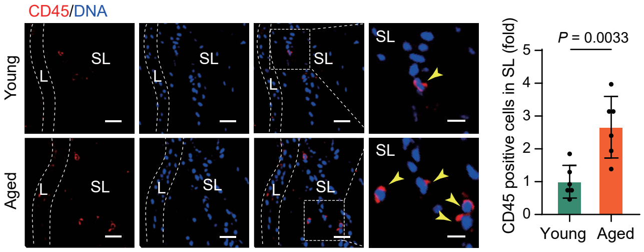
CD45/DNA
Young
Aged
L
SL
P = 0.0033
CD45 positive cells in SL (fold)
5
4
3
2
1
0
Young
Aged

Figure. S2

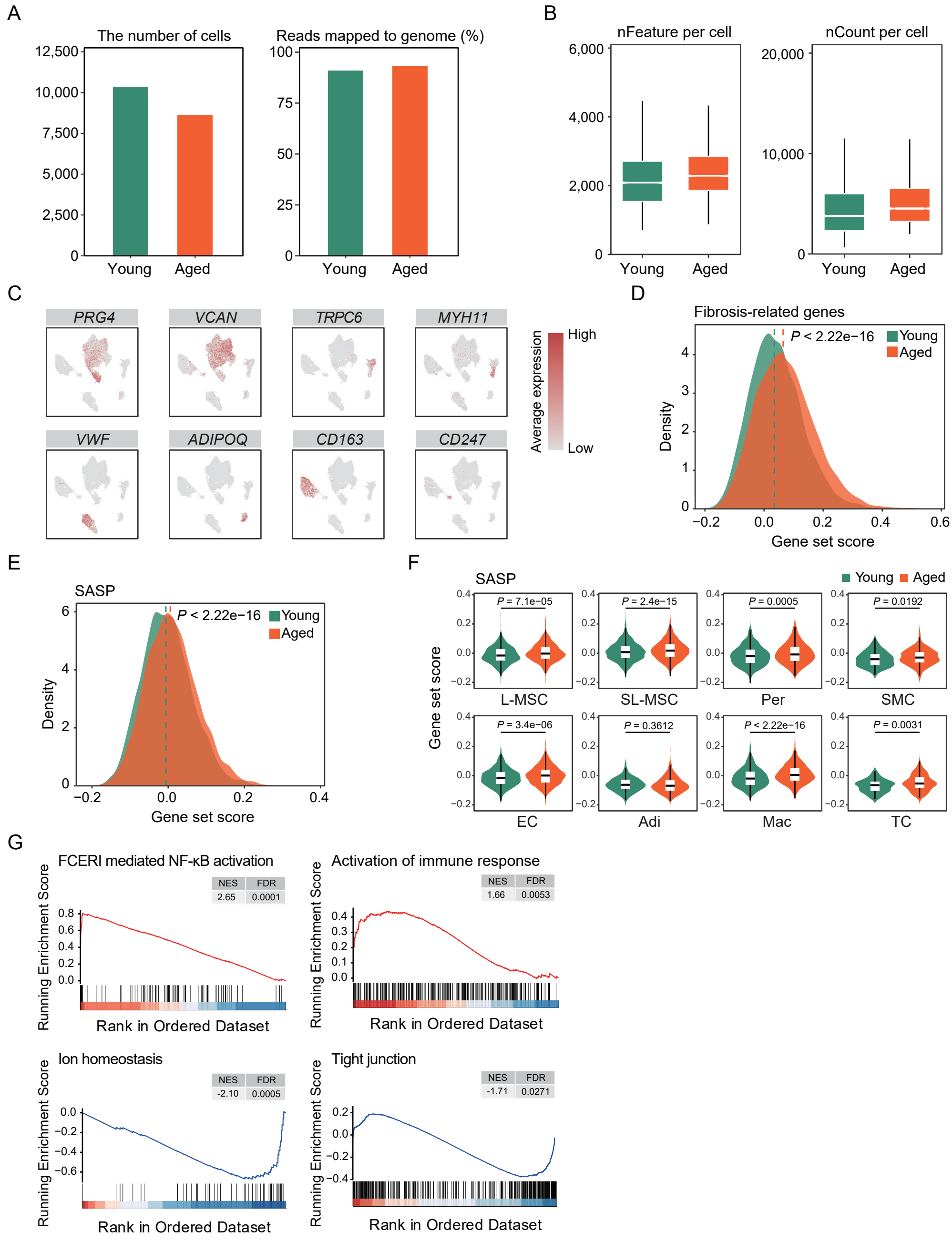
A
The number of cells
12,500
10,000
7,500
5,000
2,500
0
Young
Aged
Reads mapped to genome (%)
100
75
50
25
0
B
nFeature per cell
6,000
4,000
2,000
nCount per cell
20,000
10,000
C
PRG4
VCAN
TRPC6
MYH11
VWF
ADIPOQ
CD163
CD247
Average expression
High
Low
D
Fibrosis-related genes
P < 2.22e−16
Density
Gene set score
E
SASP
F
Gene set score
P = 7.1e−05
P = 2.4e−15
P = 0.0005
P = 0.0192
L-MSC
SL-MSC
Per
SMC
P = 3.4e−06
P = 0.3612
P < 2.22e−16
P = 0.0031
EC
Adi
Mac
TC
G
FCERI mediated NF-κB activation
NES 2.65
FDR 0.0001
Activation of immune response
NES 1.66
FDR 0.0053
Ion homeostasis
NES -2.10
FDR 0.0005
Tight junction
NES -1.71
FDR 0.0271
Running Enrichment Score
Rank in Ordered Dataset

Figure. S3

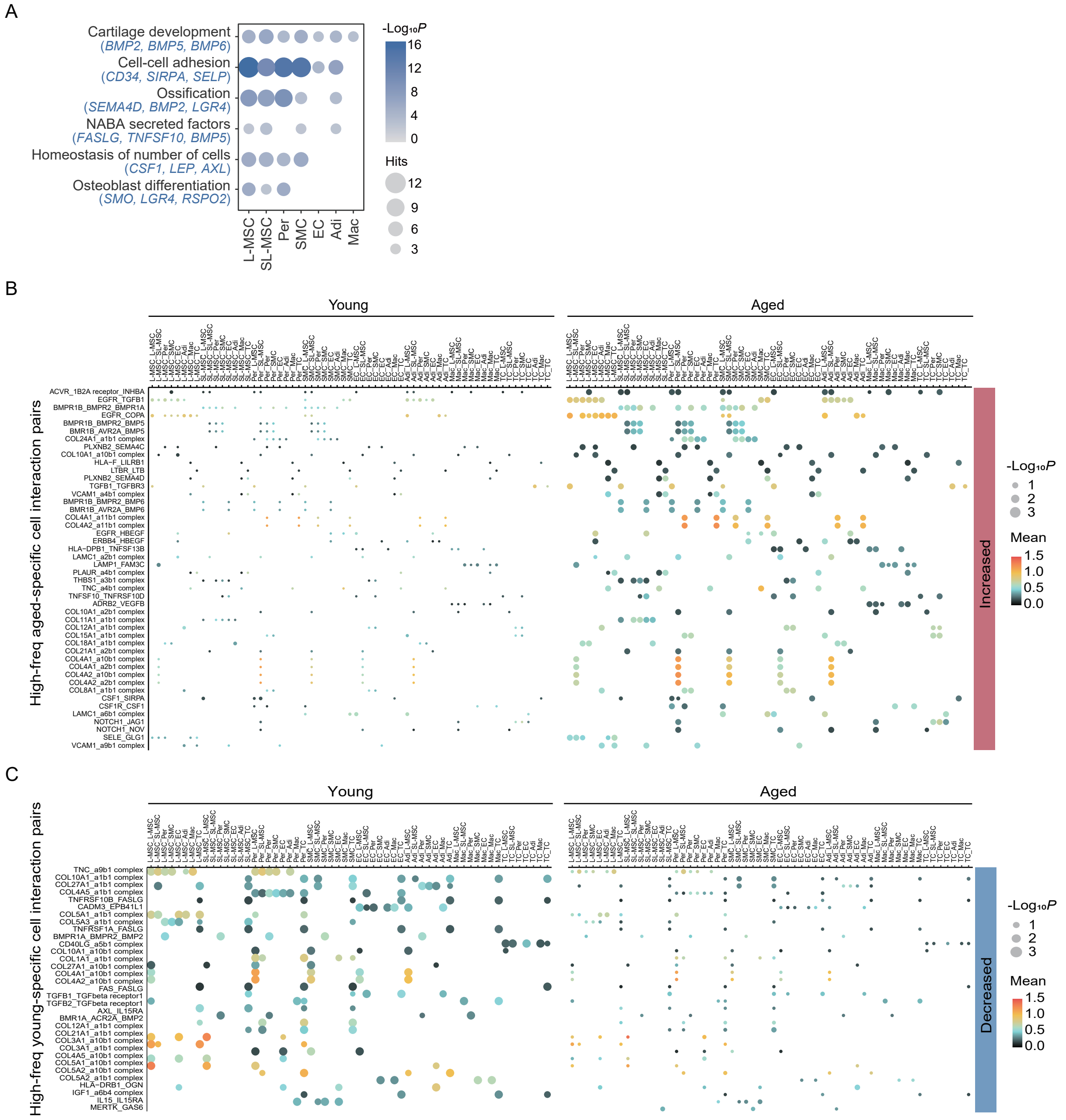
A
Cartilage development
(BMP2, BMP5, BMP6)
Cell-cell adhesion
(CD34, SIRPA, SELP)
Ossification
(SEMA4D, BMP2, LGR4)
NABA secreted factors
(FASLG, TNFSF10, BMP5)
Homeostasis of number of cells
(CSF1, LEP, AXL)
Osteoblast differentiation
(SMO, LGR4, RSPO2)
L-MSC
SL-MSC
Per
SMC
EC
Adi
Mac
-Log10P
16
12
8
4
0
Hits
12
9
6
3
B
Young
Aged
High-freq aged-specific cell interaction pairs
ACVR_1B2A receptor_INHBA
EGFR_TGFB1
BMPR1B_BMPR2_BMPR1A
EGFR_COPA
BMPR1B_BMPR2_BMP5
BMR1B_AVR2A_BMP5
COL24A1_a1b1 complex
PLXNB2_SEMA4C
COL10A1_a10b1 complex
HLA−F_LILRB1
LTBR_LTB
PLXNB2_SEMA4D
TGFB1_TGFBR3
VCAM1_a4b1 complex
BMPR1B_BMPR2_BMP6
BMR1B_AVR2A_BMP6
COL4A1_a11b1 complex
COL4A2_a11b1 complex
EGFR_HBEGF
ERBB4_HBEGF
HLA−DPB1_TNFSF13B
LAMC1_a2b1 complex
LAMP1_FAM3C
PLAUR_a4b1 complex
THBS1_a3b1 complex
TNC_a4b1 complex
TNFSF10_TNFRSF10D
ADRB2_VEGFB
COL10A1_a2b1 complex
COL11A1_a1b1 complex
COL12A1_a1b1 complex
COL15A1_a1b1 complex
COL18A1_a1b1 complex
COL21A1_a2b1 complex
COL4A1_a10b1 complex
COL4A1_a2b1 complex
COL4A2_a10b1 complex
COL4A2_a2b1 complex
COL8A1_a1b1 complex
CSF1_SIRPA
CSF1R_CSF1
LAMC1_a6b1 complex
NOTCH1_JAG1
NOTCH1_NOV
SELE_GLG1
VCAM1_a9b1 complex
Increased
-Log10P
1
2
3
Mean
1.5
1.0
0.5
0.0
C
Young
Aged
High-freq young-specific cell interaction pairs
TNC_a9b1 complex
COL10A1_a1b1 complex
COL27A1_a1b1 complex
COL4A5_a1b1 complex
TNFRSF10B_FASLG
CADM3_EPB41L1
COL5A1_a1b1 complex
COL5A3_a1b1 complex
TNFRSF1A_FASLG
BMPR1A_BMPR2_BMP2
CD40LG_a5b1 complex
COL10A1_a10b1 complex
COL1A1_a1b1 complex
COL27A1_a10b1 complex
COL4A1_a10b1 complex
COL4A2_a10b1 complex
FAS_FASLG
TGFB1_TGFbeta receptor1
TGFB2_TGFbeta receptor1
AXL_IL15RA
BMR1A_ACR2A_BMP2
COL12A1_a1b1 complex
COL21A1_a1b1 complex
COL3A1_a10b1 complex
COL3A1_a1b1 complex
COL4A5_a10b1 complex
COL5A1_a10b1 complex
COL5A2_a10b1 complex
COL5A2_a1b1 complex
HLA−DRB1_OGN
IGF1_a6b4 complex
IL15_IL15RA
MERTK_GAS6
Decreased
-Log10P
1
2
3
Mean
1.5
1.0
0.5
0.0

Figure. S4

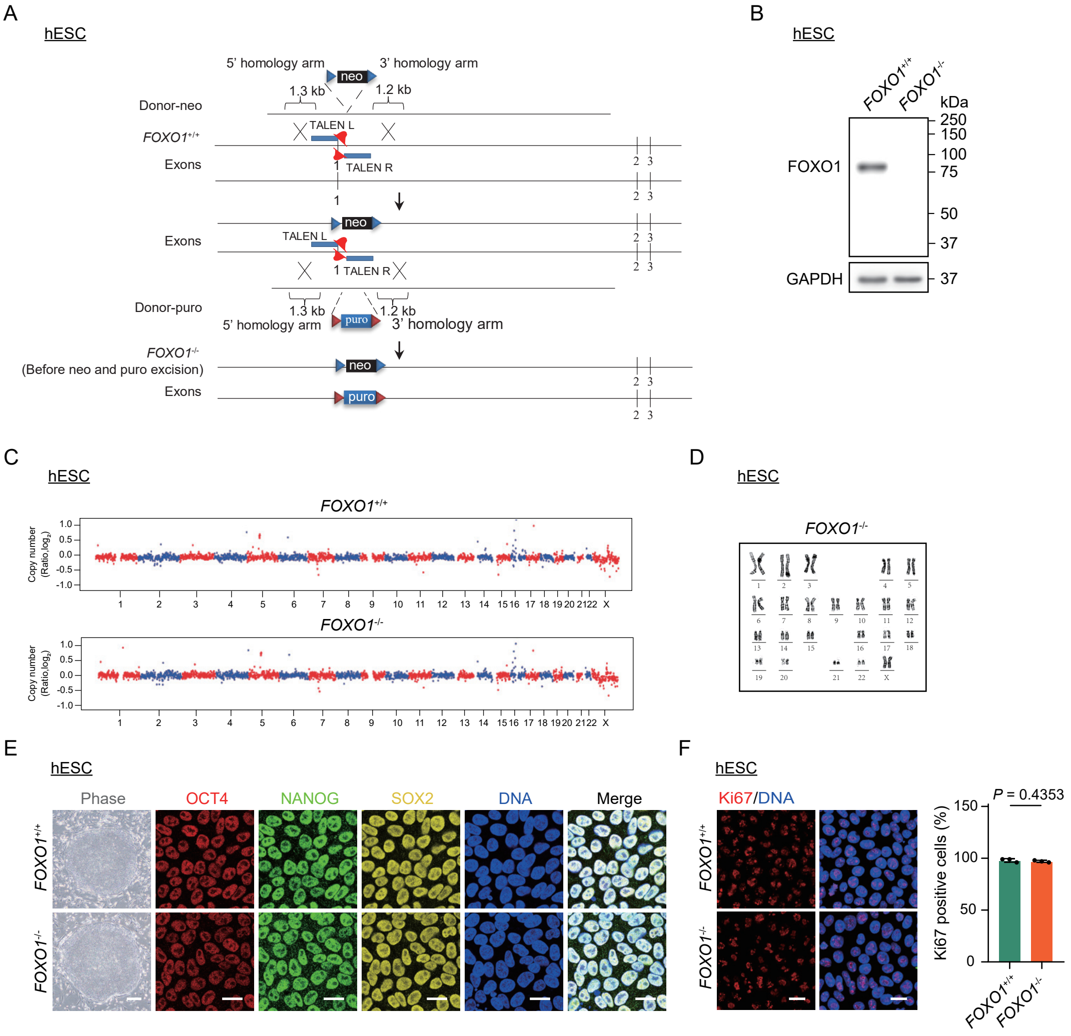
A
hESC
5' homology arm
3' homology arm
neo
1.3 kb
1.2 kb
Donor-neo
TALEN L
FOXO1+/+
Exons
TALEN R
Donor-puro
puro
FOXO1-/-
(Before neo and puro excision)
B
hESC
FOXO1+/+
FOXO1-/-
kDa
FOXO1
GAPDH
C
hESC
Copy number (Ratio,log2)
D
hESC
E
hESC
Phase
OCT4
NANOG
SOX2
DNA
Merge
F
hESC
Ki67/DNA
P = 0.4353
Ki67 positive cells (%)

Figure. S5

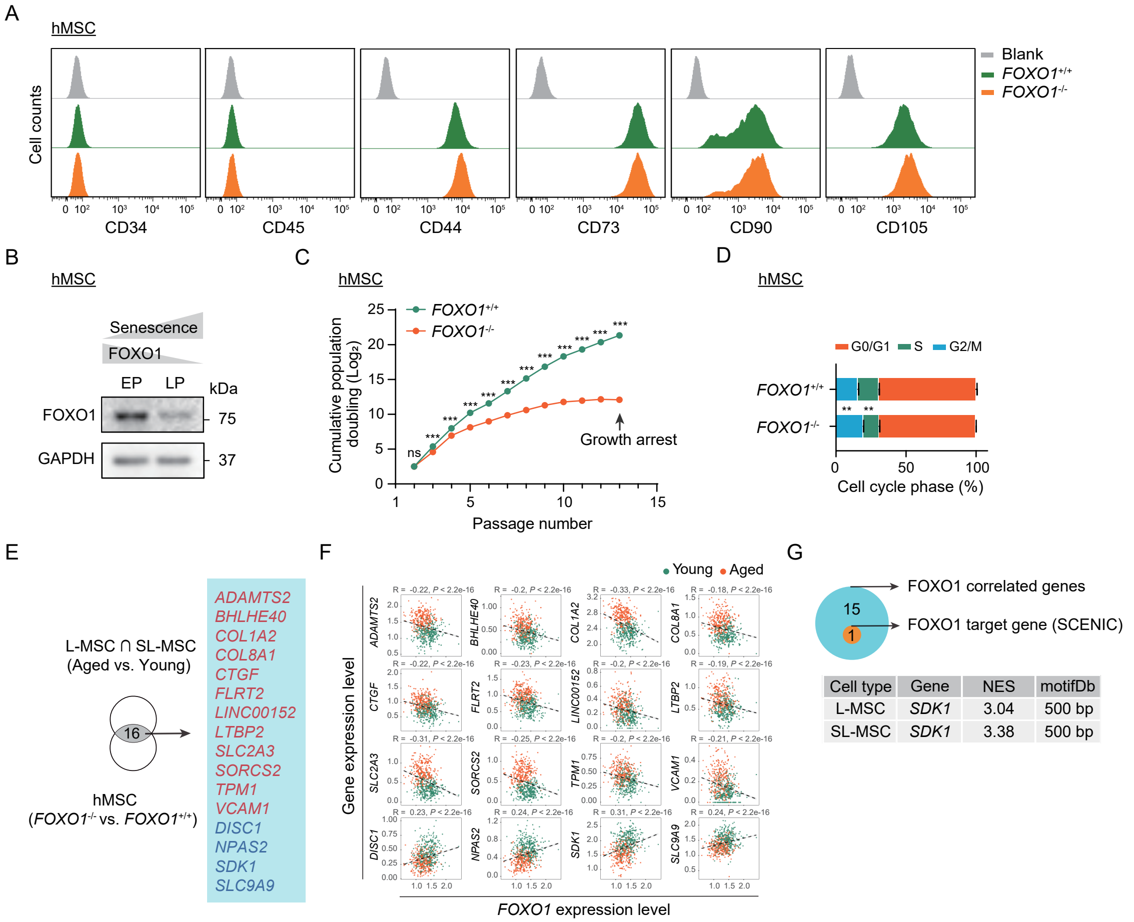
A
hMSC
Cell counts
Blank
FOXO1+/+
FOXO1-/-
CD34
CD45
CD44
CD73
CD90
CD105
B
hMSC
Senescence
FOXO1
EP
LP
kDa
FOXO1
75
GAPDH
37
C
hMSC
FOXO1+/+
FOXO1-/-
Cumulative population doubling (Log2)
Passage number
ns
Growth arrest
D
hMSC
G0/G1
S
G2/M
FOXO1+/+
FOXO1-/-
Cell cycle phase (%)
E
L-MSC ∩ SL-MSC
(Aged vs. Young)
16
hMSC
(FOXO1-/- vs. FOXO1+/+)
ADAMTS2
BHLHE40
COL1A2
COL8A1
CTGF
FLRT2
LINC00152
LTBP2
SLC2A3
SORCS2
TPM1
VCAM1
DISC1
NPAS2
SDK1
SLC9A9
F
Young
Aged
Gene expression level
FOXO1 expression level
G
15
1
FOXO1 correlated genes
FOXO1 target gene (SCENIC)
Cell type | Gene | NES | motifDb
L-MSC | SDK1 | 3.04 | 500 bp
SL-MSC | SDK1 | 3.38 | 500 bp
